# Pathologic tau conformer ensembles induce dynamic, liquid-liquid phase separation events at the nuclear envelope

**DOI:** 10.1186/s12915-021-01132-y

**Published:** 2021-09-09

**Authors:** Sang-Gyun Kang, Zhuang Zhuang Han, Nathalie Daude, Emily McNamara, Serene Wohlgemuth, Laura Molina-Porcel, Jiri G. Safar, Sue-Ann Mok, David Westaway

**Affiliations:** 1 Centre for Prions and Protein Folding Diseases, University of Alberta, 204 Brain and Aging Research Building, Edmonton, AB T6G 2 M8 Canada; 2 Department of Biochemistry, University of Alberta, Edmonton, AB Canada; 3 Neurology Department, Hospital Clinic, IDIBAPS, Barcelona, Spain; 4 Department of Neurology and Pathology, Case Western Reserve University, Cleveland, OH USA; 5 Division of Neurology, University of Alberta, Edmonton, AB Canada

**Keywords:** Tauopathy, Focal tau pathology, Liquid-solid phase transition, Nuclear-cytoplasmic transport, Transgenic mouse

## Abstract

**Background:**

The microtubule-associated protein tau forms aggregates in different neurodegenerative diseases called tauopathies. Prior work has shown that a single P301L mutation in tau gene, *MAPT*, can promote alternative tau folding pathways that correlate with divergent clinical diagnoses. Using progressive chemical denaturation, some tau preparations from the brain featured complex transitions starting at low concentrations of guanidine hydrochloride (GdnHCl) denaturant, indicating an ensemble of differently folded tau species called conformers. On the other hand, brain samples with abundant, tangle-like pathology had simple GdnHCl unfolding profile resembling the profile of fibrillized recombinant tau and suggesting a unitary conformer composition. In studies here we sought to understand tau conformer progression and potential relationships with condensed liquid states, as well as associated perturbations in cell biological processes.

**Results:**

As starting material, we used brain samples from P301L transgenic mice containing tau conformer ensembles that unfolded at low GdnHCl concentrations and with signatures resembling brain material from P301L subjects presenting with language or memory problems. We seeded reporter cells expressing a soluble form of 4 microtubule-binding repeat tau fused to GFP or YFP reporter moieties, resulting in redistribution of dispersed fluorescence signals into focal assemblies that could fuse together and move within processes between adjacent cells. Nuclear envelope fluorescent tau signals and small fluorescent inclusions behaved as a demixed liquid phase, indicative of liquid-liquid phase separation (LLPS); these droplets exhibited spherical morphology, fusion events and could recover from photobleaching. Moreover, juxtanuclear tau assemblies were associated with disrupted nuclear transport and reduced cell viability in a stable cell line. Staining for thioflavin S (ThS) became more prevalent as tau-derived inclusions attained cross-sectional area greater than 3 μm^2^, indicating (i) a bipartite composition, (ii) in vivo progression of tau conformers, and (iii) that a mass threshold applying to demixed condensates may drive liquid-solid transitions.

**Conclusions:**

Tau conformer ensembles characterized by denaturation at low GdnHCl concentration templated the production of condensed droplets in living cells. These species exhibit dynamic changes and develop in vivo, and the larger ThS-positive assemblies may represent a waystation to arrive at intracellular fibrillar tau inclusions seen in end-stage genetic tauopathies.

**Supplementary Information:**

The online version contains supplementary material available at 10.1186/s12915-021-01132-y.

## Background

Intracellular inclusions of microtubule-associated protein tau are the pathological hallmark of tauopathies including Alzheimer’s disease and some forms of frontotemporal lobar degeneration (FTLD) [1, 2]. Tau is encoded by the *MAPT* gene and is expressed mainly in neurons as six different isoforms, depending on neuronal type and maturation stage [2–4]. Tau stabilizes and maintains the architecture of microtubules and axonal integrity of neurons, in which tau is in a dynamic equilibrium between a microtubule-bound and cytoplasmic free state [2, 5]. The conformational change of monomeric soluble tau into other conformers that include hyperphosphorylated oligomers, paired helical filaments, and fibrillized tau is thought to contribute to neuronal toxicity and cell death [1–3]. We recently reported that the *MAPT*-P301L mutations affecting tau with 4 microtubule-binding repeats generates distinct tau conformers as appraised by conformation-dependent immunoassay and conformational stability assays (CSAs) using guanidine hydrochloride (GdnHCl) denaturant [6]. The diverse and evolving repertoire of tau conformers was postulated as the origin of the neuropathological and biochemical heterogeneity of FTLD with tau immunoreactive inclusions (FTLD-tau) [1, 6]. Moreover, in frontotemporal dementia, a neurological diagnosis associated with the neuropathological diagnosis of FTLD, conformational stabilities were correlated with different clinical disease variants. However, the cellular events that draw a line from protein conformation to neurological dysfunction are not well understood.

Nuclear localization of tau has been observed and suggested to facilitate genome surveillance under conditions of cellular stresses [7]. In neurodegenerative disorders including frontotemporal dementia, Alzheimer’s disease, Huntington’s disease, Parkinson disease, and amyotrophic lateral sclerosis, disruption of nuclear-cytoplasmic transport has been proposed as a toxic mechanism mediated by abnormally aggregated proteins [8–14]. Nuclear pore complexes, which are one of the largest macromolecular assemblies found in eukaryotic cells, reside in the nuclear envelope and mediate nuclear-cytoplasmic transport of various nuclear proteins and RNAs [15–17]. These cellular components, as well as lamin proteins that contribute to the lamina of the nuclear envelope, may have an intrinsic jeopardy to accumulating damage in chronological aging as they have remarkably low rates of turnover [18]. For tau, it is accepted that alterations in the physiological properties resulting from post-translational modifications, conformational changes, and/or pathogenic mutations, can lead to mislocalization and formation of inclusions in neuronal cell bodies [1, 2]. More recently, there has been a focus upon whether tau inclusions cause an impairment of nuclear-cytoplasmic transport, following from sequestration of nucleoporins [14] and nuclear deformation [19] (both in vitro and in vivo), incurring toxic consequences.

It is possible that these newly documented changes in tau and nuclear proteins may intersect with discoveries in a third axis of work concerning membraneless organelles formed by a phase separation process. Membraneless organelles have been highlighted as active bioreactors regulating cell signaling, protein synthesis, and various biological reactions against environmental stresses [20–23]. Membraneless organelles need to be assembled as functional condensed droplets and disassembled by quality control processes within a confined biological time scale if irreversible conformational changes are to be avoided [24, 25]. In amyotrophic lateral sclerosis/frontotemporal dementia, mutations in low complexity domains and/or RNA recognition motifs are responsible for neurotoxicity by disrupting the dynamics of physiological membraneless organelles [22]. Pathogenic mutations in transactive response DNA-binding protein 43 (TDP-43, also known as TARDBP), heterogeneous nuclear ribonucleoprotein A1 (hnRNPA1) and fused in sarcoma (FUS) alter biophysical properties of membraneless organelles from reversible metastable liquid condensates to irreversible persistent fibrous aggregates [24, 26–28]. Recent studies of tau have also revealed a propensity to undergo liquid-liquid phase separation (LLPS) [5, 29–35].

In experiments described herein, we used starting material obtained from the brains of aged, low-expresser P301L transgenic mice [36] bred onto a congenic genetic background [37]. Drawing upon four types of assays, we used brain fractions inferred to contain mixed pathogenic conformers of phospho-tau species [37], rather than samples with a predominance of fibrillar tau forms characterized by a single melting transition in chemical denaturation studies [6]. These brain fractions produced juxtanuclear tau aggregates in a reporter assay; here we show that liquid phase condensation of tau occurred in living cells subjected to seeding with this brain material. Irregular condensed tau assemblies produced in this manner sequestered nucleoporins from nuclear pore complexes and hence reduced cell viability by virtue of impeding vital nuclear trafficking. Moreover, the staining properties of juxtanuclear larger structures in seeded cells are indicative of laminar/concentric arrangements; these data point to a possible sequence of events in the evolution of pathogenic tau conformers, extending prior conclusions drawn from histopathological and chemical analyses of aging cohorts of the P301L transgenic mice [6, 37].

## Results

### Droplet-like behavior of tau inclusions

**Fig. 1:**
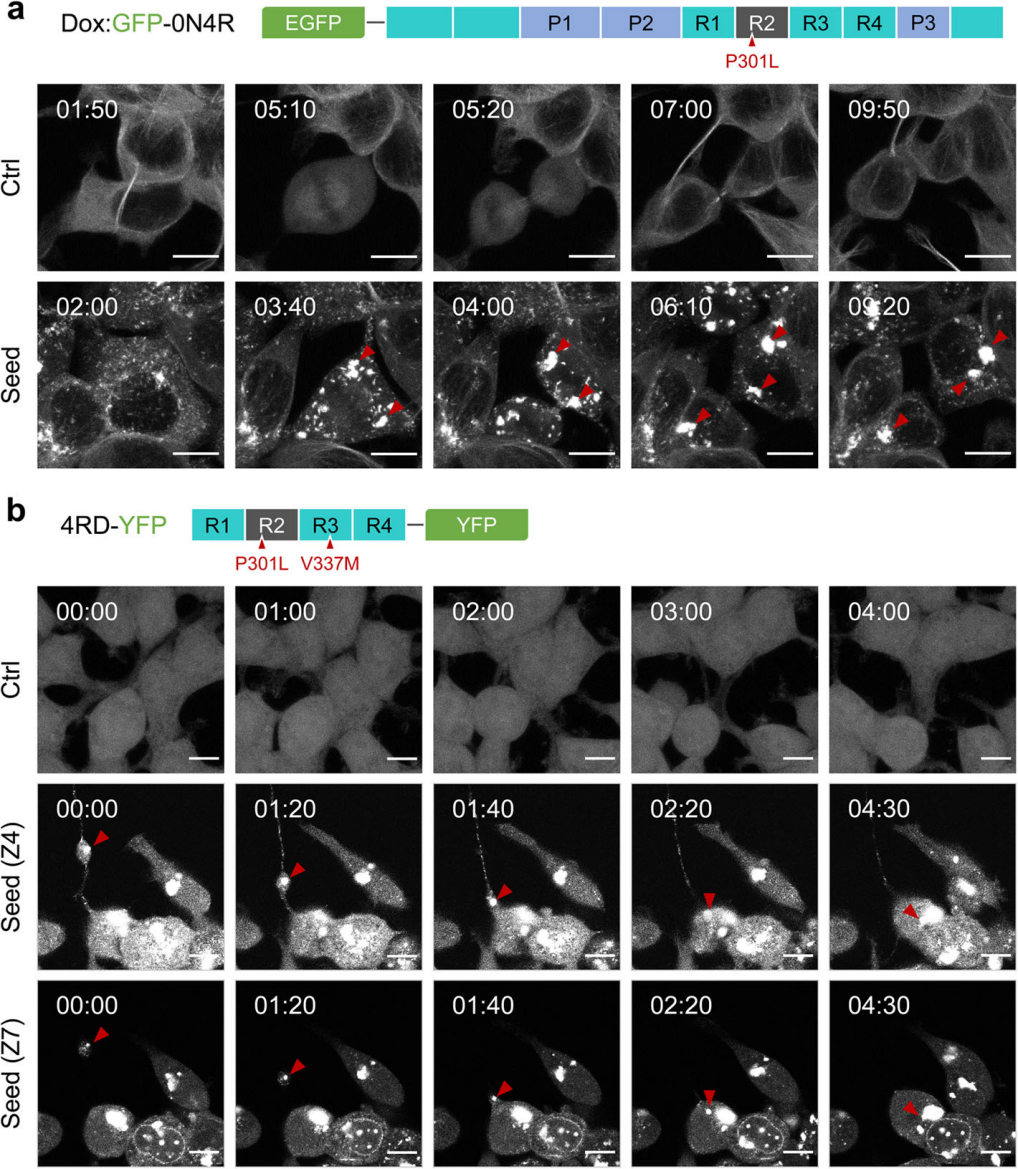
Dynamic aspects of tau inclusion formation. Doxycycline-inducible GFP-0N4R (**a**) and 4RD-YFP (**b**) tau reporter cell lines express human tau with aggregation prone mutations P301L and P301L/V377M, respectively. The cells were seeded with brain fractions including seed-competent pathogenic tau derived from TgTau^P301L^ mice with a CSA Type 2 denaturation profile for sarkosyl-insoluble tau. Droplet-like movement of intracellular tau inclusions (**a**) and transfer through a tunneling nanotube-like membrane extension (**b**) were analyzed by time-lapse imaging of the seeded cells for 5 h (5 min/frame for 60 frames). Z4 and Z7 are different focal planes with the 3-μm interval. Scale bars, 10 μm

To investigate the biological attributes of tau aggregates, S1 brain fractions containing seed-competent tau conformers derived from aged TgTau^P301L^ mice [37, 38] were transduced into two recipient cell lines based on different configurations of tau four-repeat domain (4RD) reporter molecules. Firstly, we established reporter cells from a single-cell clone of human embryonic kidney 293 cells (HEK293) expressing a doxycycline-inducible green fluorescent protein (GFP) fused with full-length human tau (0N4R) with an aggregation prone mutation (P301L) (Dox:GFP-0N4R P301L) (Fig. 1a). Secondly, we also used HEK293-derived DS1 reporter cells [37, 39, 40]), a clonal isolate that expresses yellow fluorescent protein (YFP) fused with a 4RD region of human tau with two FTLD-MAPT mutations (P301L/V337M), (4RD-YFP P301L/V377M) (Fig. 1b). The GFP-0N4R form of tau (a 66-kDa species) was observed in the cytoplasm as expected, while the 4RD-YFP tau (a 45-kDa species) yielded signals spread throughout the cell body to include the nucleus (Fig. 1a, b); the latter may be due to passive macromolecular diffusion through nuclear pore complexes which decrease beyond a 30–60 kDa size threshold [17].

Live cell imaging of tau-seeded cells was undertaken to investigate dynamic aspects of tau inclusion formation. These analyses revealed that small inclusions fuse together to make larger inclusions (Fig. 1a, Supplementary Movies 1 and 2), this being consistent with the previous report that the dynamic structure of tau aggregates undergo “fusion” and “fission” in stable cell lines expressing full-length human tau T40 (2N4R) carrying the P301L mutation with a GFP tag (T40/P301L-GFP) [41]. We also noted cell-to-cell spread of tau where tau inclusions moved to adjacent cells through tunneling nanotubes and fused with others, which produced larger inclusions (Fig. 1b, Supplementary Movie 3 to 5). Since transferring tau inclusions are compact and suspended in three-dimensional cellular space [34], their detection is dependent upon the position of image acquisition. For instance, depending upon focal planes, tau inclusions moved into an adjacent cell by way of tunneling nanotubes or seemed to be discerned as a droplet being phagocytosed by a neighboring cell (Fig. 1b, Supplementary Movies 4 and 5). In the former case, the structure of tunneling nanotubes spreading tau inclusions were clearly visible in between cells located approximately 10–20 μm from each other but were not visible in clumped cells (Supplementary Figure 1, Supplementary Movies 6, 7, 8 and 9). Further research is required as to whether the transferred materials can initiate tau nucleation in unseeded recipient cells. It has been reported that attachment of a GFP tag to a recombinant tau RD—but not the full-length molecule—affects the formation of paired helical filament-like fibrils under cell-free conditions [42]. However, here the same types of tau inclusion signatures were found in cells (GFP-0N4R and 4RD-YFP cell lines), using either N- or C-terminal reporter moieties indicating (i) that pathogenic tau seeds have an intrinsic potential to trigger coalescence of both the repeated domain and the full-length tau in our assays and (ii) that these results do not represent the idiosyncratic behavior of a single-cell line.

### Tau concentration and phase transition

**Fig. 2:**
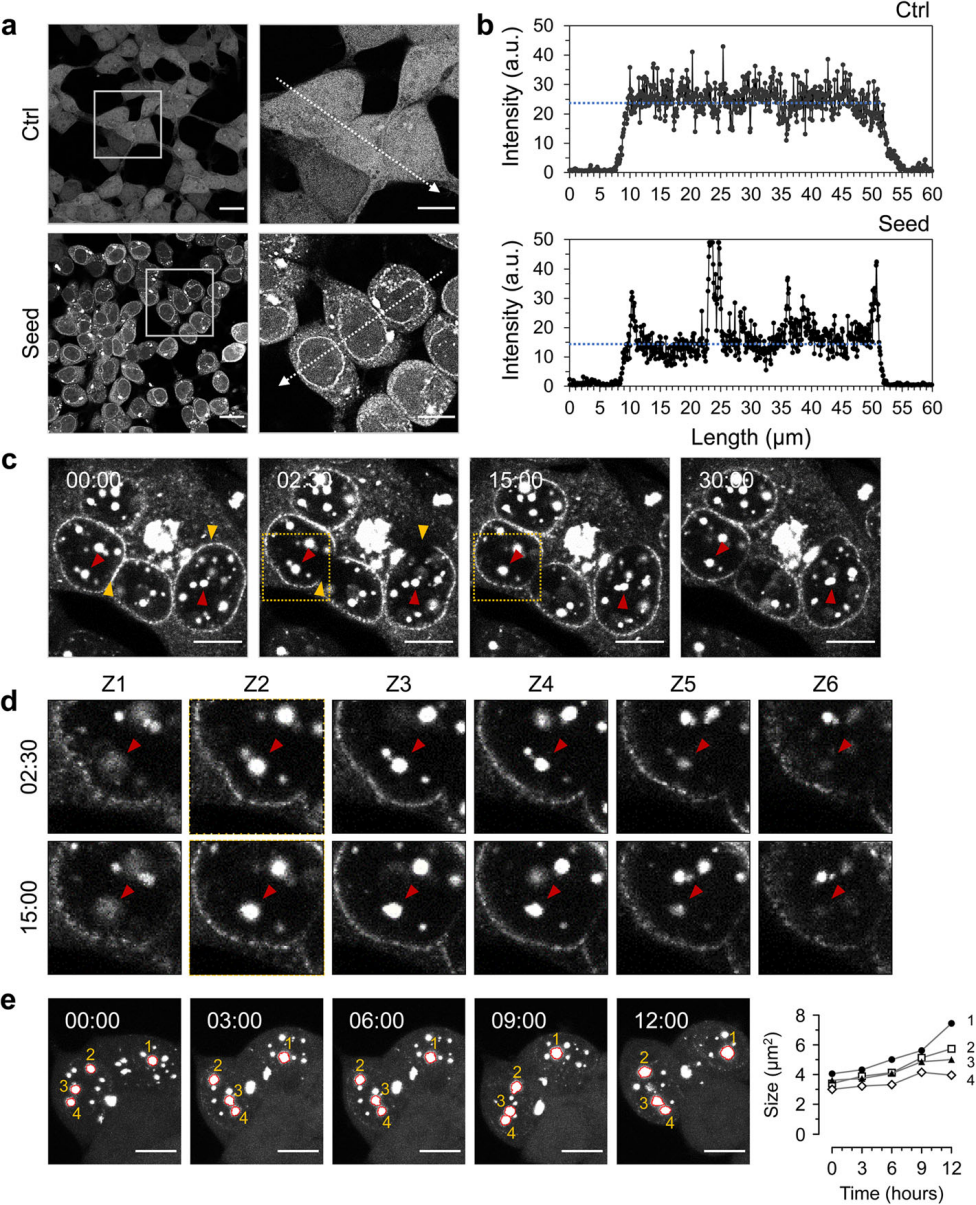
Tau concentration on the nuclear envelope and droplet-like tau inclusions. **a, b** Tau condensation occurred by pathogenic tau seeding. 4RD-YFP tau reporter cells were seeded as per Fig. 1 and imaged (**a**). Plot profiling analysis of tau signal intensities were conducted along the arrows with a length of 60 μm (**b**). a.u., arbitrary units. Scale bar, 20 μm and 10 μm in the boxed images. **c** Fluorescence recovery after photobleaching (FRAP) analysis for tau inclusions on the nuclear envelope and droplet fusions in ES1 cells. Nuclear envelope tau signals were photobleached (yellow arrowheads) at the indicated time point and then time-lapse images were obtained every 30 s for 30 min. Droplet-shaped tau inclusions fused together and became larger inclusions (red arrowheads). Scale bar, 10 μm. **d** Different focal plane images of the boxed areas in (**c**) at indicated time points. Z1 to Z6 are depths of field from bottom to top with 1-μm intervals. Arrowheads indicate tau inclusions fused into one bigger droplet. **e** Droplet fusions in the seeded cells were monitored by time-lapse imaging for 12 h (10 min/frame for 72 frames) and the increases in cross-sectional area (μm^2^) were measured every 3 h. Scale bar, 10 μm

We next sought evidence for the in vivo formation of a liquid state of tau. Confirming and extending previous analyses [6], dispersed tau-YFP signals were sequestered to tau inclusions by pathogenic tau seeding (Fig. 2a), presenting as heterogeneous fluorescent morphologies and with a repeated occurrence of juxtanuclear signals (Supplementary Figure 2a). Quantification of image pixels demonstrated that concentration of tau on the nuclear envelope occurred by the seeding reaction. The average intensity in the cell bodies of tau reporter cells was 23.7 ± 0.2 arbitrary units (a.u.), while the seeded cells showed 14.1 ± 0.1 a.u. (Fig. 2b). In a stable cell clone harboring tau inclusions, designated “ES1” and reflecting a single-cell clone established by limiting dilution of the seeded reporter cells, nuclear envelope tau signals were rapidly regained (within 15 min) in fluorescence recovery after photobleaching (FRAP) analysis (Fig. 2c, Supplementary Movie 10). Notably, different focal plane images revealed that tau inclusions showed liquid droplet-like movements and fused together (Fig. 2d). Droplet fusion events and increases in cross-sectional area are detected in both types of reporter cells (i.e., Dox:GFP-0N4R P301L and 4RD-YFP cell lines) following pathogenic tau seeding (Fig. 2e, Supplementary Figure 2b and c, Supplementary Movies 11, 12, 13 and 14). In sum, these properties align with common criteria for defining a phase-separated structure under live cell conditions, namely spherical morphology, fusion events, and recovery from photobleaching [21, 43].

**Fig. 3:**
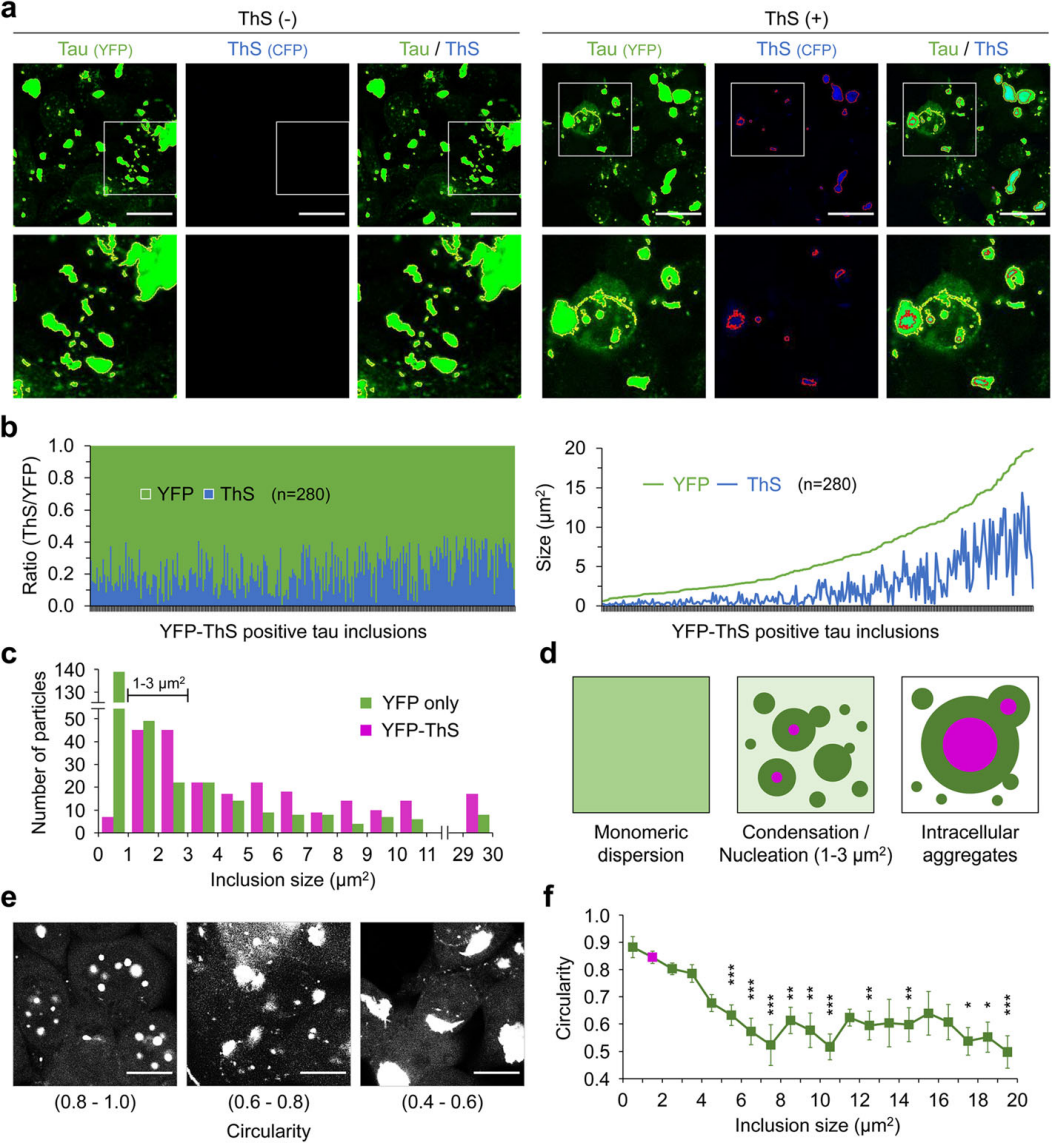
Tau assemblies with a concentric structure. **a** Intracellular aggregates in the ES1 clonal line harboring tau inclusions. Cells were stained with Thioflavin S (ThS), which increased its fluorescence upon binding to the intermolecular β-sheet structure present in the protein aggregates. Tau (outlined in yellow) and ThS (outlined with red) signals were obtained using YFP and CFP channels, respectively, in the absence and presence of ThS staining. Scale bar, 20 μm. **b** Size measurement of YFP-ThS double-positive tau inclusions observed in **a**. A total of 280 particles from 8 different areas (160 μm^2^ for each, see Supplementary Figure 3) were analyzed and presented as a ratio of ThS to YFP (left) and the area of ThS and YFP within each inclusion (right). **c** Size distributions of YFP-only positive (*n* = 331) versus YFP-ThS double-positive (*n* = 317) tau inclusions; the majority of YFP-only positive tau inclusions were smaller than 1 μm^2^ (*n* = 139). **d** Schematic illustrating condensation of monomeric dispersed tau into demixed liquid droplets (dark green). The center and right-hand panels show concentric laminar structures for droplets with ThS-positive interiors, as inferred from the data in panel **c**. Primary nucleation of tau (magenta) occurs in droplets with a size of 1–3 μm^2^. Tau aggregates grow by recruiting further condensed tau droplets and droplets including small aggregates. **e, f** Morphometric analysis of the YFP-ThS double-positive tau inclusions shown in **b**. **e** Representing images of circularities. Scale bar, 10 μm. **f** The mean circularities of the individual inclusions in different size groups were compared to that of the 1–2 μm^2^ size group, which is dominant in frequency (colored in magenta, *n* = 45). Error bars represent SEM. *p < 0.05, **p < 0.01 and ***p < 0.001 by one-way ANOVA with Tukey’s multiple comparison test

To further confirm LLPS of tau in response to seeding by exogenous pathogenic tau, ES1 cells exhibiting a heterogeneous repertoire of tau inclusion phenotypes were stained with thioflavin S (ThS), which shows an increase in the emission of fluorescent signals upon by binding to fibrillar assemblies [30, 44]. ThS staining readily visualized amorphous and juxtanuclear inclusions in these analyses, but—crucially—not nuclear envelope inclusions nor small speckles (Fig. 3a, Supplementary Figure 3). Intriguingly, particle size plotted against fluorescent signal for the YFP and ThS double-positive inclusions revealed that the YFP-associated area under the curve was always wider than ThS area (Fig. 3b), thus indicating that ThS-positive forms of aggregated tau could have a surrounding milieu of condensed tau existing in a liquid state. In line with this, most of ThS signals overlapped with YFP as determined by Manders’s colocalization coefficients; ThS (M1) = 0.956, YFP (M2) = 0.681 (Fig. 3a). In this regard, we noted that particle distribution in these experiments demonstrated a dramatic changeover in fluorescent properties dependent upon size (Fig. 3c). Thus, for tau inclusions of less than 1 μm^2^ in size, YFP-only particles dominated by a ratio of 28:1 over ThS-positive species. After this size, the ratio change approached unity, with absolute counts of particles peaking in the size range 1 to 3 μm^2^. For inclusion sizes ≥ 4 μm^2^, the ratio of YFP-only to ThS-positive species changed to define a consistent overabundance of ThS-positive species.

Taken together, these data indicate that the dispersed soluble form of cellular tau concentrated and underwent LLPS under conditions initiated by tau seeding. Importantly, the primary nucleation of tau aggregates, which has been inferred from observations under in vitro conditions of molecular crowding [5, 29, 30, 45], was demonstrated here within living cells seeded with pathogenic tau (Fig. 3d). For condensed tau droplets, above a size range of 1 μm^2^ and increasing up to 30 μm^2^ (Fig. 3c), the positivity for both YFP and ThS signals indicated a bipartite structure. The decline in circularity, a morphometric shape descriptor of the double-positive inclusions dependent upon its size, provided further support for the concept of laminated droplet structures (Fig. 3e, f).

### Decline in cell viability of ES1 cells

**Fig. 4:**
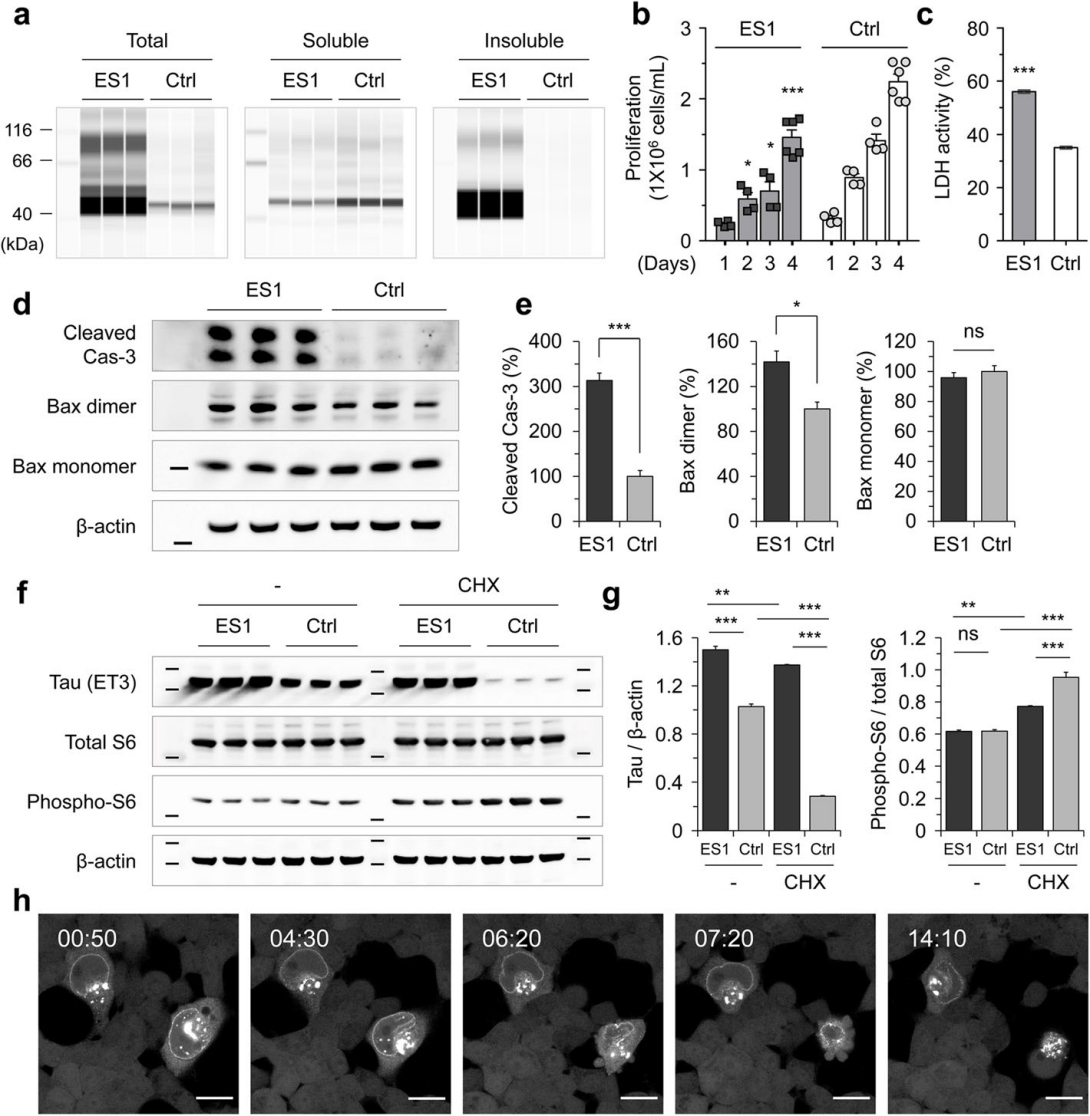
Tau-mediated reduction in cell viability of ES1 cells. **a** Sedimentation of Triton X-100 insoluble tau. ES1 and control cell lines (the reporter cells) were lysed in PBST and sedimented at 100,000×*g* for 1 h. The amount of tau in soluble and insoluble fractions was analyzed using capillary western with anti-tau mAb (ET3) (*n* = 3). **b** Proliferation of ES1 cells was determined by counting viable cells at the indicated time points (*n* = 4). **c** LDH activity in conditioned media of ES1 was measured as an indicator of cell death at 3 days post splitting (*n* = 4 independent cultures each with 4 technical replicates). **d** Western blot analysis of apoptotic cell death in ES1. The amount of cleaved caspase 3 (Cas-3) and dimerized Bax were analyzed in ES1 and control lines (*n* = 3). **e** Intensity measurement of the western results in **d**. Intensities were normalized to those of β-actin. Error bars represent SEM. **f** Western blot analysis of the 40S ribosomal protein S6. **g** Intensity measurement of the western results in **f**. Translational rates in ES1 and control cells were appraised by the ratio of the phosphorylated ribosomal protein S6 to total S6 in the presence and absence of cycloheximide (CHX, at 20 μg/mL for 16 h) (*n* = 3). Error bars represent SEM. One-way ANOVA with Tukey’s multiple comparison test. **h** Time-lapse imaging of 4RD-YFP tau reporter cells seeded with pathogenic tau as per Fig. 1. The cells including nuclear envelope tau inclusions underwent regulated cell death with nuclear deformation. Cells were imaged every 10 min for 15 h. Scale bar, 20 μm. *p < 0.05, **p < 0.01 and ***p < 0.001 in comparison with the controls

We then explored whether the condensed tau was associated with impairments in cellular physiology. Sedimentation analysis revealed that ES1 cells contained mostly insoluble forms of tau, whereas non-seeded reporter cells (4RD-YFP P301L/V377M) had entirely soluble tau (Fig. 4a). ES1 cells showed a decrease in proliferation (Fig. 4b) and an increase in lactate dehydrogenase (LDH) activities in the conditioned media compared to the non-seeded reporter cells (Fig. 4c). These findings overlap prior reports that an elevated level of cell death was linked to the presence of tau inclusions [39, 40]. Levels of cleaved caspase 3 (Cas-3) and Bax dimers, which are apoptotic cell death markers [46, 47], were also higher in ES1 than in un-transduced reporter cells (Fig. 4d, e). Six different sublines of ES1 were established to assess any effects of ongoing, divergent evolution of protein aggregates that could affect endpoint measures, i.e., “strain divergence” in the terminology of the prion field. However, those clones were not obviously distinguishable from the parental ES1, exhibiting the same size of protease-resistant core for tau following limited proteolytic digestion (Supplementary Figure 4). Oligomeric and filamentous tau conformers have been reported to hinder protein synthesis in cultured cell models [48, 49]. Considering translational stress as a potential confounding event in the cloned ES1 line, we performed western blot analysis of the 40S ribosomal protein S6. The analysis failed to reveal significant changes in the ratio of the phosphorylated ribosomal protein S6 to total S6, indicating that the intracellular condensation process occurring in ES1 cells did not affect the translational machinery (Fig. 4f, g). On the other hand, time-lapse imaging analysis revealed apoptosis-like death of seeded 4RD-YFP reporter cells with nuclear envelope and juxtanuclear tau inclusions, as characterized by nuclear collapse and formation of apoptotic bodies (Fig. 4h, Supplementary Movie 15).

### Defects of nuclear envelope in ES1 cells

**Fig. 5:**
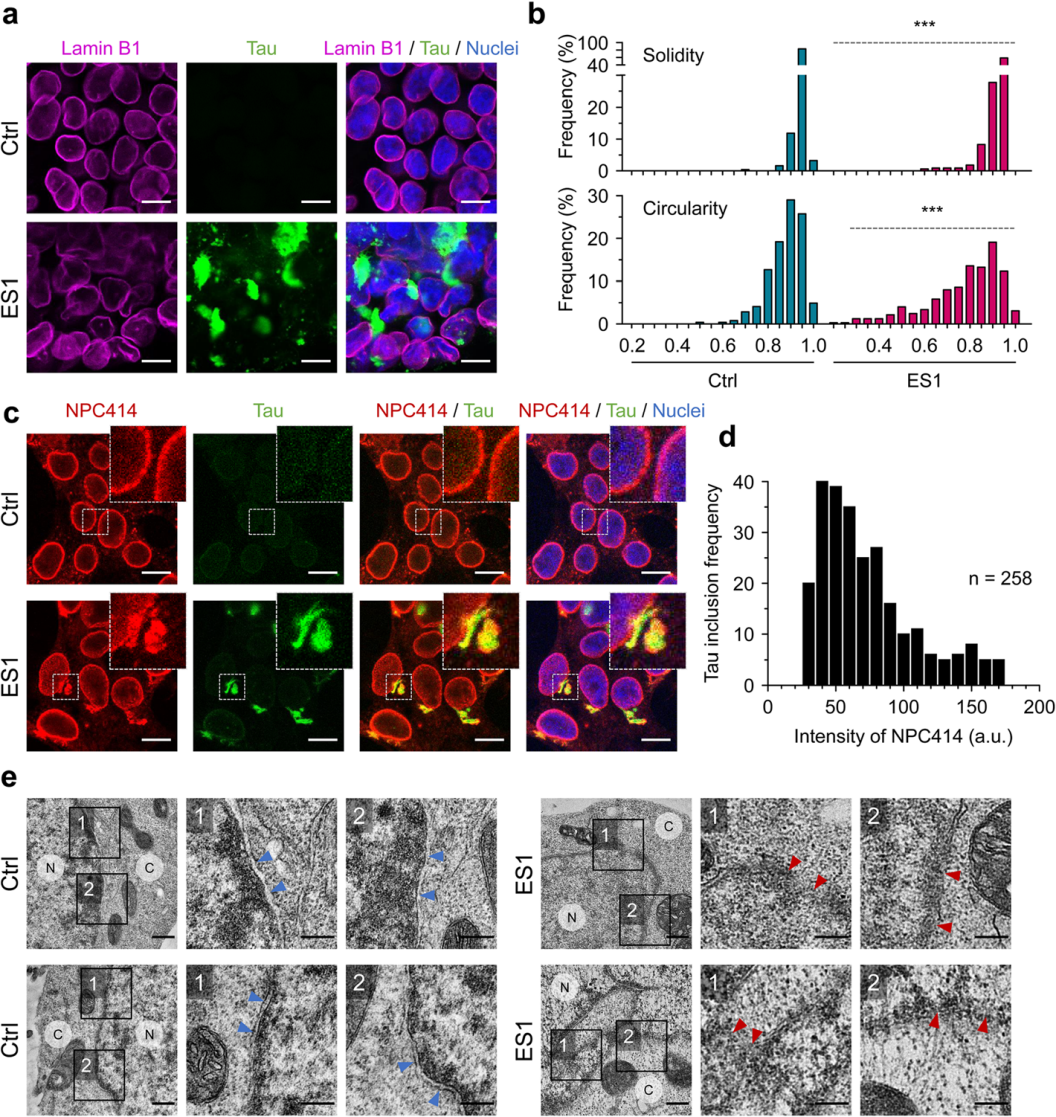
Mislocalization of nucleoporins and nuclear membrane rupture in ES1 cells. **a**–**d** Nuclear deformation and mislocalization of nucleoporins. 4RD-YFP tau reporter (Ctrl) and ES1 cells were fixed. Lamin B1 (**a**) and nucleoporins (**c**) were probed and visualized with fluorescent conjugated secondary antibodies. Lamin B1 in magenta; nucleoporins in red; tau in green; nuclei were counterstained with hoechst 33342 (blue). Scale bar, 10 μm. **b** Morphometric shape descriptors, solidity and circularity, were measured along the nuclear margins (lamin B1 staining) to appraise nuclear deformations in ES1 line (*n* = 325) compared to controls (*n* = 245). Nuclei from 8 different areas (160 μm^2^ for each, see Supplementary Figure 5). **d** Colocalization of nucleoporins (using anti-nucleoporins antibody, NPC414) with tau inclusions were determined by measuring intensity of NPC414 signals within tau inclusions over 1 μm^2^ in size. Total 258 inclusions from 6 different areas (160 μm^2^ for each) were analyzed. The level of colocalization was presented as the frequency of tau inclusion per unit intensity of NPC414. a.u., arbitrary unit. **e** TEM analysis of control (left) (*n* = 2) and ES1 cells (right) (*n* = 6) showed a disruption of the double membrane architecture of the nuclear envelope (also see Supplementary Figure 6c). Arrowheads (blue, normal; red, ruptured) indicate nuclear envelopes. C, cytoplasm; N, nucleoplasm. Scale bar, 500 nm and 250 nm in the boxed images

Building on the above observation of nuclear tau condensation (Figs. 2a, c, 3a, 4h) and reports by others [14, 19], we considered whether demixed nuclear envelope tau might interfere with the functionality of nuclear pore complexes, and thereby cause disrupted nuclear-cytoplasmic transport, noting that nuclear pore complexes reside in the nuclear envelope and mediate bidirectional nuclear-cytoplasmic transport of molecules essential for cell proliferation and survival [50, 51]. Under certain conditions of tauopathy, tau binds to nucleoporins [14], which are the main components of the nuclear pore complexes and are embedded in the central lumen of nuclear pore complexes. Immunocytochemistry of nuclear lamin B1 revealed a deformation and/or protrusions of the nuclear envelope (Fig. 5a, Supplementary Figures 5, and 6a). To give greater precision about nuclear abnormalities, individual nuclei from ES1 and control cells were analyzed using morphometric descriptors along the nuclear margins including circularity, solidity, roundness, and cross-sectional area. The morphometric shape analysis of ES1 nuclei showed significantly larger variabilities in solidity, circularity, and cross-sectional area compared to controls, indicating a nuclear distortion occurred in ES1 cells (Fig. 5b, Supplementary Figure 6b). Mislocalization of nucleoporins using the monoclonal antibody, NPC414, detecting conserved Phe and Gly-rich repeats on nucleoporins 62, 90, and 152 was determined in ES1 cells (Fig. 5c). The mislocalized nucleoporins were appraised by measuring the mean pixel intensity of NPC414 in individual tau inclusions with a size over 1 μm^2^ (Fig. 5d), overlapping some previous observations that tau mutations can cause microtubule-mediated deformation of nuclei, as seen in postmortem analyses of tissues [19]. The nuclear envelope itself consists of the inner and outer nuclear membranes, which are separated by the perinuclear space [16, 52]. Transmission electron microscopy (TEM) confirmed that the structural integrity of the double-layered nuclear envelope was ruptured in ES1 cells versus controls (Fig. 5e, Supplementary Figure 6c). These data support a view that condensed tau on the nuclear envelope in ES1 cells triggers the separation of nucleoporins from nuclear pore complexes, disrupts molecular trafficking across the nuclear envelope, and thereby contributes to cellular dysfunction that may then trigger regulated cell death pathways.

### Impairment of nuclear-cytoplasmic compartmentalization in ES1 cells

**Fig. 6:**
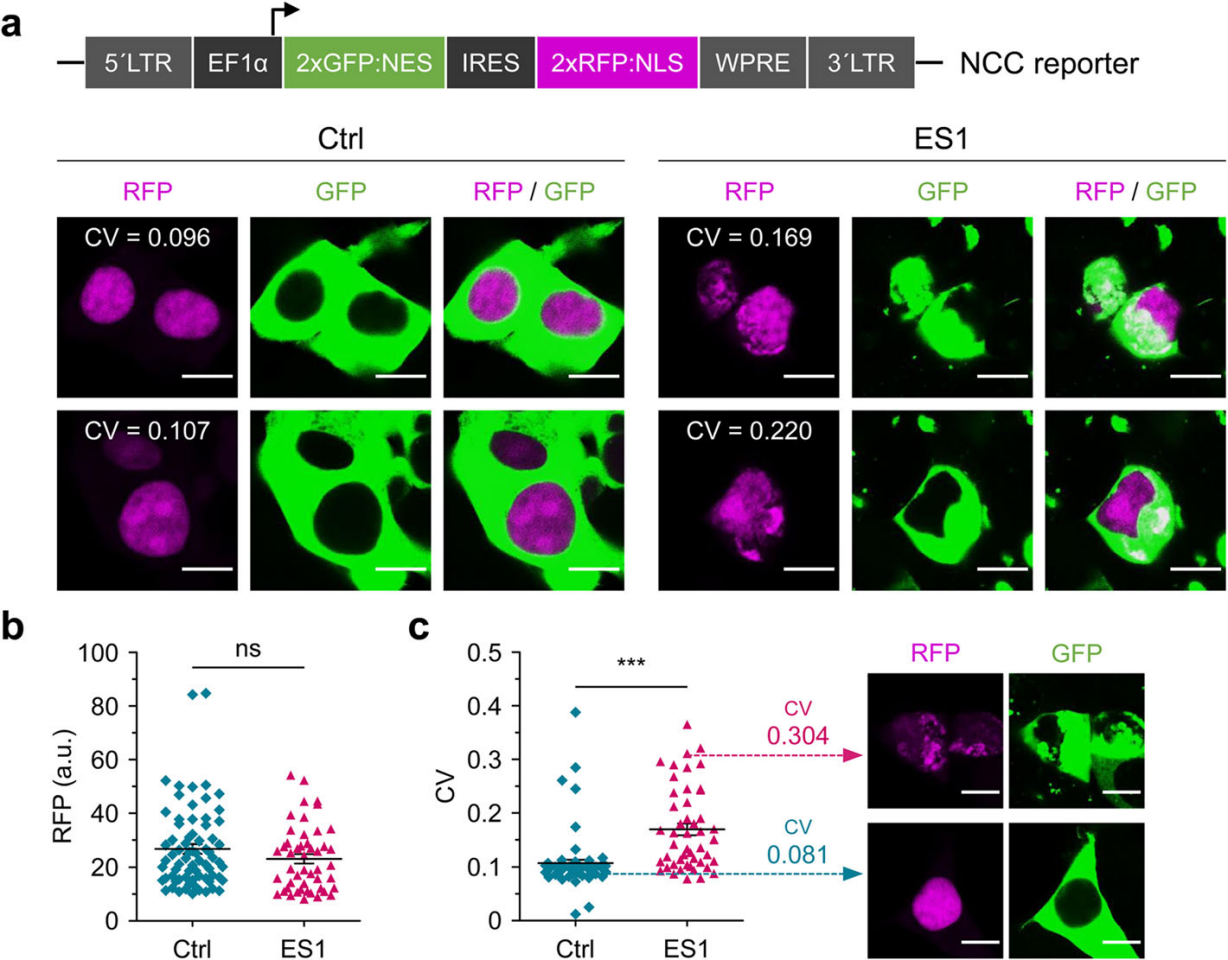
Disruption of nuclear-cytoplasmic compartmentalization in ES1 cells. **a** Schematic illustration of nuclear-cytoplasmic compartmentalization (NCC) reporter construct (top). 4RD-YFP tau reporter (Ctrl) and ES1 cells transiently transfected with the NCC reporter (bottom). Control cells exhibited segregated fluorescent signals, green cytoplasm and red nuclei (colored in magenta), whereas red fluorescence signals were colocalized with cytoplasmic green fluorescence in ES1 cells, indicating a defect in NCC. CV, the coefficient of variation of the mean pixel intensity of red fluorescence; RFP, red fluorescent protein; GFP, green fluorescent protein. Scale bar, 10 μm. **b, c** The mean pixel intensity of RFP (**b**) and the CV (**c**) in the transfected reporter (*n* = 71) and ES1 cells (*n* = 48). Cells from 8 different areas, respectively (160 μm^2^ for each, see Supplementary Figure 7). a.u., arbitrary unit. Scale bar, 10 μm

For dynamic analyses of nuclear-cytoplasmic transport, tau reporter cells (4RD-YFP P301L/V377M) and the ES1 line were transfected with an additional type of reporter construct, a plasmid encoding two proteins indicating the status of nuclear-cytoplasmic compartmentalization (NCC) events. The NCC reporter plasmid (pLVX-EF1alpha-2xGFP:NES-IRES-2xRFP:NLS) has been used by others [14, 19, 53]; it encodes a nuclear export signal (NES) fused to a green fluorescent protein (GFP) and a nuclear localization signal (NLS) fused to a red fluorescent protein (RFP) under the control of a human elongation factor-1α (EF-1α) promoter and with an internal ribosome entry sequence (IRES) located between the two open reading frames (Fig. 6a) [53]. Corresponding tau reporter cells exhibited a segregated arrangement of the fluorescent signals; GFP localized in the cytoplasm and RFP localized in nuclei. Although YFP signals of tau inclusion in ES1 cells hindered the analysis of NES-GFP compartmentalization, increased levels of local RFP signals in both nuclei and cytoplasm indicated an impairment of NCC (Fig. 6a, Supplementary Figure 7). Analyses of mean pixel intensities in the RFP channel showed no significant difference between the ES1 and control cells transfected with the NCC reporter construct (Fig. 6b), while the degree of variation appraised by the coefficient of variation (CV) was significantly high in ES1 cells, indicating an uneven distribution of RFPs (Fig. 6c).

**Fig. 7:**
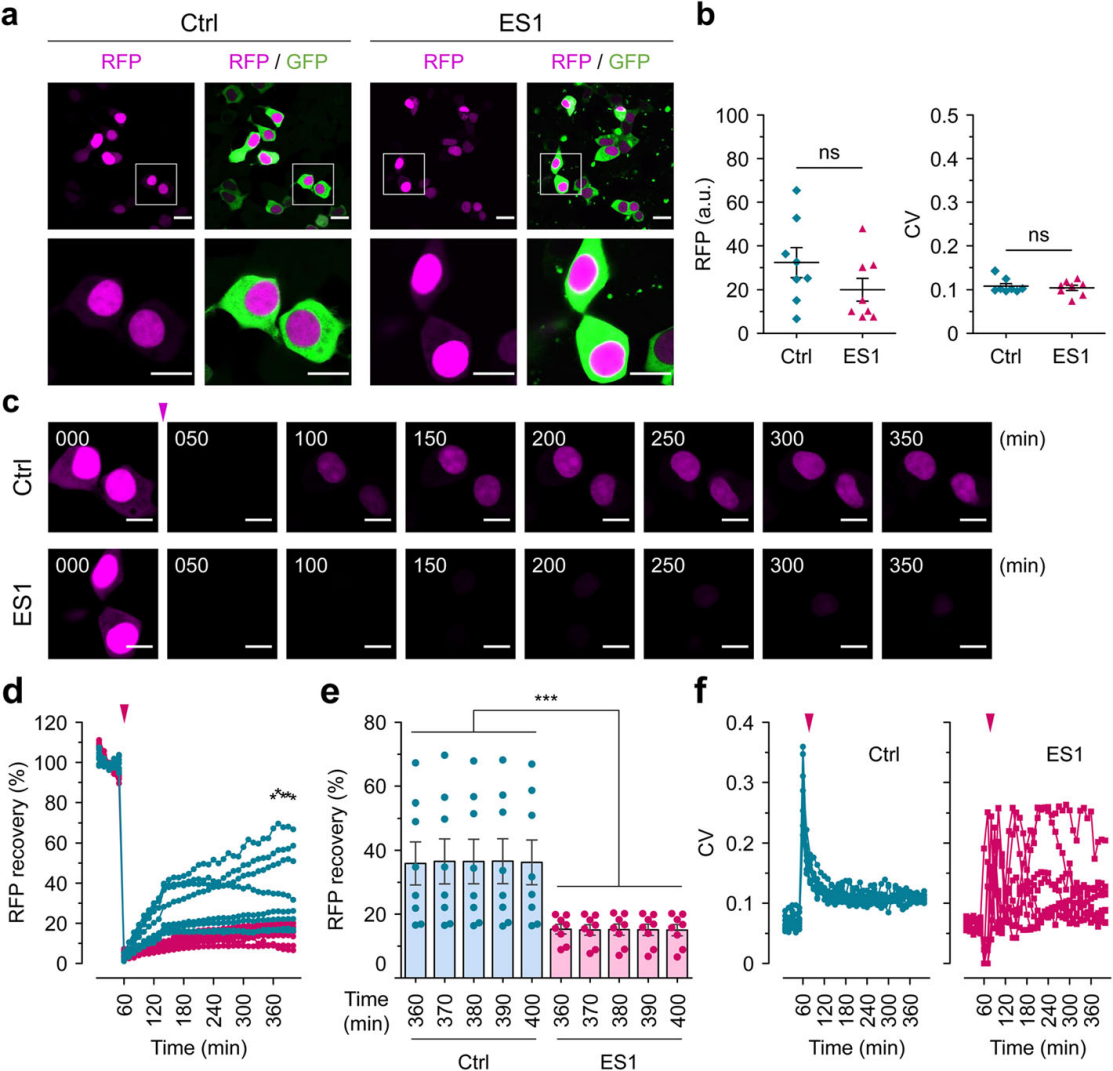
Interference with nuclear trafficking. **a** Normal nuclear-cytoplasmic compartmentalization (NCC) observed in both 4RD-YFP tau reporter (Ctrl) and ES1 cells transiently transfected with the NCC reporter. Scale bars, 20 μm and 10 μm in the boxed images. **b** The mean pixel intensity of the red fluorescent signals (left) and the coefficient of variation (CV) (right) in the transfected reporter (*n* = 8) and ES1 cells (*n* = 8) shown in **a**. RFP, red fluorescent protein; a.u., arbitrary unit. **c** Time-lapse imaging of FRAP analysis. Red fluorescence signals (colored in magenta) were completely photobleached and then images of signal recovery were obtained every 10 min for 6 h. The arrowhead in magenta indicates the point of photobleaching applied. Scale bar, 10 μm. **d** Real-time measurements of fluorescence recovery of nuclear red signals in tau reporter (*n* = 8) and ES1 cells (*n* = 8) shown in **a**. The means of individual time points were compared to one another. *p < 0.05 by one-way ANOVA with Tukey’s multiple comparison test. **e** The signal recoveries in the last five time points revealed a significant decline in the nuclear trafficking of RFP in ES1 cells compared to the controls. Error bars represent SEM. ***p < 0.001 in comparison with the controls. **f** The variation in the CV of the mean pixel intensities measured in the FRAP analysis from **d**

Several ES1 cells transfected with the NCC reporter showed normal GFP/RFP NCC signals based on the mean pixel intensity and the low CV values (Figs. 6c, 7a, b), but degrees of impaired NCC in other cells were related to the severity of tau-mediated nuclear envelope damage. ES1 transfected cells harboring the pLVX-EF1alpha-2xGFP:NES-IRES-2xRFP:NLS plasmid and showing the anticipated segregated pattern of the fluorescent signals were then subjected to FRAP analysis. Since the emission spectra of GFP encoded in the NCC reporter and YFP fluorophore fused to the tau repeat domain overlap, we restricted ourselves to the use of RFP signals for FRAP analyses. RFP signals in the entire images were photobleached, such that nuclear RFP signals appearing de novo in the field of view must derive from newly synthesized molecules. Five initial time-lapse images were taken as points of reference, with subsequent recovery of signal in the RFP channel measured every 10 min thereafter for 6 h (Fig. 7c, Supplementary Movies 16 and 17). Nuclear RFP signals in tau reporter cells recovered within an hour after photobleaching up to 19.0 ± 2.9% and reached up to 36.3 ± 7.0% (mean ± SEM); the ES1 cells on the other hand showed a slower recovery, with only 8.2 ± 0.6% signal at 1 h and a final attained value of 15.0 ± 1.8% (mean ± SEM) (Fig. 7d, e; the mean recovery of individual RFP signals from control and ES1 cells were compared to each other at the indicated time points). Interestingly, higher variability (CV) was evident for nuclear RFP signals in ES1 cells during the course of FRAP analysis, indicating perturbed nuclear transport (Fig. 7f). These observations are consistent with defects in the selective nuclear envelope permeability seen in induced pluripotent stem cell (iPSC)-derived neurons with IVS10 + 16 and P301L *MAPT* mutations [19], and in primary neurons treated with high molecular weight Alzheimer’s disease brain fractions containing tau [14].

### Nuclear distortion in human FTLD-tau cases

**Fig. 8:**
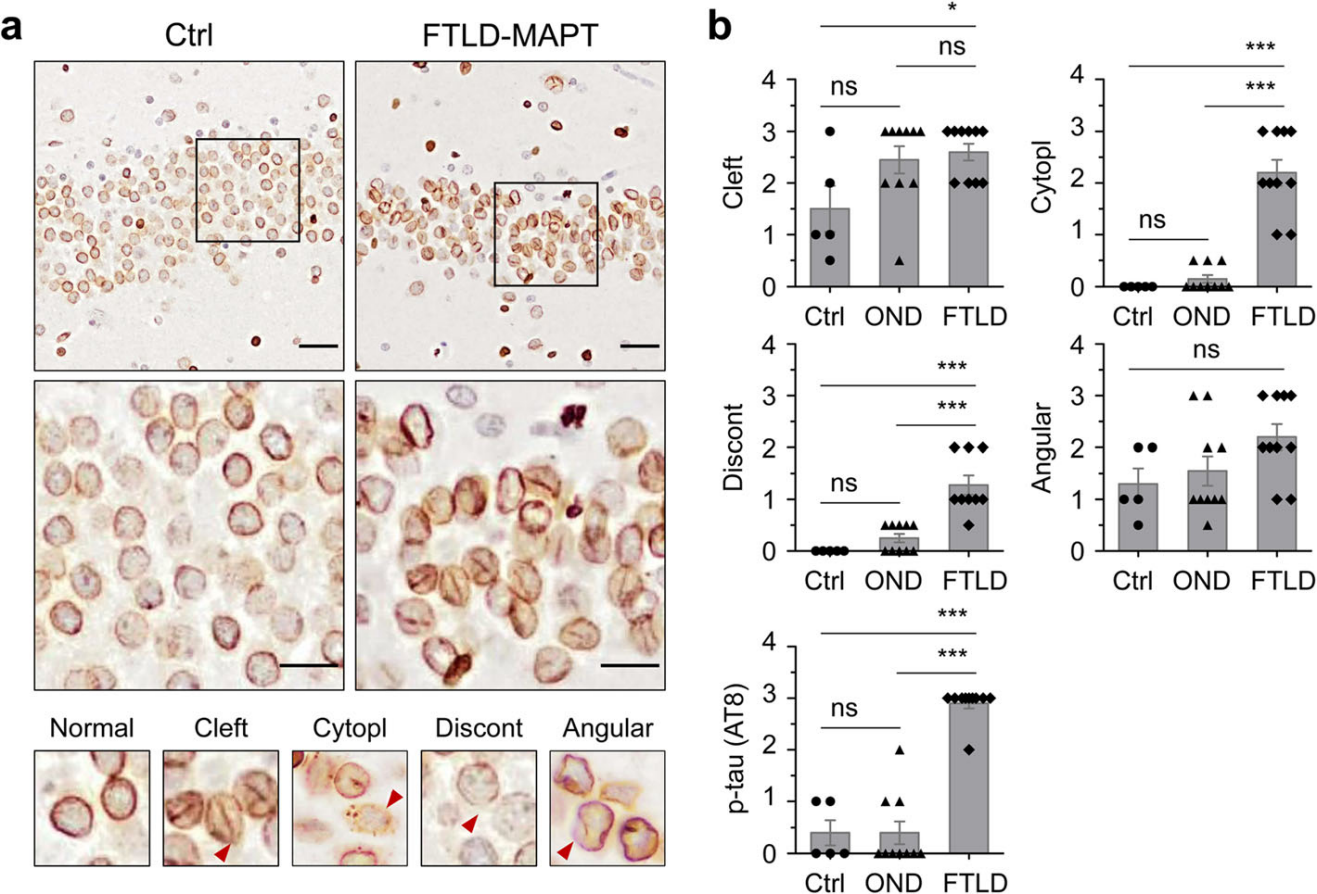
Nuclear deformation in FTLD-MAPT patients. **a** Distortion of neuronal nuclear envelopes was determined in the dentate gyrus of FTLD-MAPT patients using lamin B1 staining for nuclear lamina (upper panels). Ctrl, non-neurological disease. Lower panels: higher magnification views to show representative nuclear morphologies including Cleft, nuclear cleft; Cytopl, cytoplasmic granular lamin B stain; Discont, discontinuous nuclear edge; Angular, angled nuclear envelope. **b** Assessment of nuclear deformation in FTLD-MAPT (FTLD) (*n* = 10 subjects), non-neurological disease (controls, Ctrl) (*n* = 5), and other neurodegenerative diseases (OND) (*n* = 10) including Alzheimer’s disease (AD), frontotemporal dementia with progranulin mutations (*GRN*), amyotrophic lateral sclerosis (ALS), and amyotrophic lateral sclerosis and frontotemporal dementia (ALS/FTD). Lesions of the dentate gyrus obtained from 20 to 30 field images were scored on 3-point scale by two observers (see also “Methods” and Table 1). p-tau (AT8), phosphorylated tau detected using the monoclonal antibody, AT8. *p < 0.05 and ***p < 0.001 by one-way ANOVA with Tukey’s multiple comparison test. Scale bars, 40 μm and 20 μm in the boxed images

To investigate tau-associated nuclear deformation in FTLD-MAPT-P301L patients, nuclear lamina in postmortem cerebral cortex were probed by lamin B1 immunostaining (Fig. 8a). Our resources included brain tissue from ten Iberian FTLD-MAPT-P301L patients [54] that likely derive from a common ancestor [55]. These P301L cases, characterized previously for tau pathology and confirmed to lack confounding proteinopathies [6, 54], were augmented by a number of controls including Alzheimer’s disease cases, frontotemporal dementia with progranulin mutations, amyotrophic lateral sclerosis cases, and non-demented controls. Although other analyses have remarked upon nuclear clefts as a feature of FTLD-MAPT [19], when examining the nuclei of dentate gyrus neurons, this finding also applied to other clinical entities, being abundant within three amyotrophic lateral sclerosis cases, two progranulin mutation carriers and in one non-demented control (Fig. 8b, Table 1). Although there was a trend for lower ages at death in the P301L group, this did not reach significance and this morphological alteration was considered an age-related change that could appear in different contexts, rather than an effect restricted to FTLD-MAPT. Thus, along with other analyses [56], the hypothesis for a relationship between nuclear clefts and the specific pathogenic processes of FTLD-MAPT was not supported, prompting consideration of other nuclear alterations caused by the presence of misfolded tau isoforms. Using anti-lamin B1 and AT8 monoclonal antibodies to stain the nuclear lamina and phospho-tau (Ser202/Thr205), respectively, we assessed potential distinctions between FTLD-MAPT-P301L cases versus control samples. Discounting the occasional nuclear clefts and angular margins also present in other diseases, several distinctions were noted, which included variations in staining intensity on the margins of normally shaped nuclei (discontinuous nuclear edge) and cells with granular and apparently spherical immunostained structures in the cytoplasm (cytoplasmic granular lamin B stain) (Fig. 8b).

**Table 1:** Nuclear deformation of granular neurons in the dentate gyrus of FTLD-MAPT or other neurodegenerative disorders

Clinical diagnosis	Age at death	Sex	Nuclear morphology based on lamin B1 staining				Phospho-tau
			Cleft	Cytopl	Discont	Angular	
Control	31	M	0.5	0	0	0.5	0
Control	70	M	1	0	0 vs UK	2	0
Control	76	M	2	0	0	1	1+
Control	78	M	3	0	0	2	1+
Control	81	F	1	0	0 vs UK	1	0
AD	72	F	0.5	0	0	0.5	1+
AD	92	F	3	0.5	0.5	3	2+
*GRN*	60	M	3	0.5	0.5	2	0
*GRN*	64	M	3	0	0.5	3	1+
ALS	50	M	3	0	0	2	0
ALS	50	M	3	0	0	1	0
ALS	51	M	2	0	0	1	0
ALS	63	M	3	0	0.5	1	0
ALS	64	M	2	0	0	1	0
ALS/FTD	59	M	2	0.5	0.5	1	0
FTLD-P301L	49	M	3	2	UK	2	ND
FTLD-P301L	52	M	2	2	1	3	3+
FTLD-P301L	53	M	3	3	1	3	3+
FTLD-P301L	56	M	3	3	2	2	3+
FTLD-P301L	58	M	2	2	1	1	3+
FTLD-P301L	58	M	3	3	2	2	3+
FTLD-P301L	61	F	2	1	1	1	3+
FTLD-P301L	63	F	2	2	1	2	3+
FTLD-P301L	72	M	3	3	2	3	3+
FTLD-P301L	75	M	3	1	0.5	3	3+

Table after [54], arranged by clinical diagnosis: *control*, non-neurological diseases; *AD*, Alzheimer’s disease; *GRN*, frontotemporal dementia with mutations in the progranulin gene; *ALS*, amyotrophic lateral sclerosis; *ALS/FTD*, amyotrophic lateral sclerosis and frontotemporal dementia; *FTLD-P301L*, frontotemporal lobar degeneration with P301L mutation in the *MAPT* gene. *M*, male; *F*, female. *Cleft*, nuclear cleft; *Cytopl*, cytoplasmic granular lamin B stain; *Discont*, discontinuous nuclear edge; *Angular*, angled nuclear envelope; *Phospho-tau*, tau detected by the monoclonal antibody, AT8; *UK*, unknown; *ND*, not detected. Scoring criteria for tau deposits as per [6, 54]

## Discussion

### LLPS of tau induced by seed-competent conformers

Phase transition of tau into a demixed liquid state of tau has been reported mainly using in vitro cell-free systems with purified recombinant proteins under conditions of molecular crowding [5, 29–33, 57, 58]. Intrinsic and documented aspects of tau biology include natively disordered structure, inhomogeneous charge distribution, variable patterns of physiological and pathological phosphorylation, pathogenic mutations, and alternative splicing sites producing six different isoforms, features which might singly or collectively lead to increased acquisition of LLPS [5, 29–31]. In cultured cells, a GFP-tagged version of the longest isoform of wild-type tau (GFP-tau441) formed droplet-like accumulations in transiently transfected mouse primary cortical neurons and N2a neuroblastoma cells with high expression levels [30]. In another case, overexpression of the proline-rich domain of tau, achieved through a cryptochrome 2-based optogenetic oligomerization module, CRY2olig [59], formed heterotypic condensates with microtubule plus-end tracking proteins (i.e., end binding 1 (EB1) protein) in SH-SY5Y neuroblastoma cells [34]. Increased local concentration of aggregation prone proteins, such as pathogenic TDP-43, hnRNPA1, and FUS, has been considered to enhance protein interactions causing LLPS [20, 28, 60, 61]. In this study, a tau repeat domain/YFP fusion protein with pathogenic mutations (P301L and P301L/V337M) is stably dispersed throughout the cytoplasm and the entire cell body, without forming protein clusters. The final concentration of total tau used to seed the reporter cells, including soluble and insoluble forms in the presence of sarkosyl, was 20 ng/mL based on the estimation using conformation-dependent immunoassay [6] whereas the signal for total tau in transduced ES1 cells was approximately 8 times greater than that in non-seeded controls, as analyzed by capillary western (Fig. 4a). These data strongly suggest that there is a net, bona fide increase in tau concentration (manifest cytologically as assemblies on nuclear envelope, as well as other types of inclusions) as a result of seeding, rather than a mere redistribution effect with no net increase in tau. Moreover, the nuclear envelope tau inclusions and small droplets were found to behave as a concentrated polymeric liquid phase (as determined by a combination of FRAP, live cell imaging analysis, and staining with ThS, a reagent with a propensity to react with amyloid fibrils), with implications for the cell biology of disease pathogenesis.

### Demixed tau conformers interfere with nuclear transport

The cytotoxic effects of aggregated tau have been extensively studied in neurons, which lose proliferative capacity upon differentiation. In this study, aside from the neurotoxicity associated with an impairment of axonal transport and/or synaptic transmission (reviewed in [4, 62]), the biophysical impact of condensed tau on the nuclear trafficking was examined in the reporter cell lines. We have reported nuclear envelope tau inclusions as the predominant morphology in 4RD-YFP P301L/V377M reporter cells seeded with misfolded tau conformers (a type 2 signature in chemical denaturation assays, “CSA Type 2,” found in the brains of some aged TgTau^P301L^ mice and P301L patients [6, 37, 54]. Here, fluorescent tau deposits in immortalized 4RD-YFP (4RD-YFP P301L/V377M) or GFP-0N4R (Dox:GFP-0N4R P301L) reporter cells transduced with CSA Type 2 seeds exhibited heterogeneous morphologies, but with nuclear envelope inclusions being prominent. These inclusion morphologies persisted after the single-cell cloning to produce ES1 cells, which exhibited a lower proliferation rate and apoptotic cell loss versus non-seeded clonal controls. A commensurate decline in translational rate was considered as a contributing factor [48, 49], but was not supported in this role using the ratio of the phosphorylated ribosomal protein S6 to total S6 as a proxy measure [63]. In ES1 cells, tau-mediated toxicity by interference with nuclear transport was a slow event, manifesting only over several passages rather than causing acute cell loss; parental cells harboring tau inclusions could divide into two daughter cells (Fig. 1a, Supplementary Movies 1 and 2), or fail in division, or undergo regulated cell death (Fig. 4h, Supplementary Movie 15), possibly depending on the prolonged duration of steps for intracellular tau conformers to condense and aggregate. While we cannot exclude that pathogenic tau conformers compromise their host cells by means above and beyond altered NCC, it is also possible that these dividing cells may underestimate some toxic effects of the tau condensates because of dilution of cell contents resulting from mitosis. In post-mitotic neurons, ES1 tau conformers could coalesce on the nuclear envelope, appearing as spherical clumps, and with these assemblies being capable of undergoing liquid-solid phase transition. Similarly, glial lineage cells affected in 4R-tauopathies [1] could show diverse morphologies of tau accumulation because of the same types of transitions shown in dividing cells. For lamin B1 alterations reflecting changes in nuclear function and architecture as well as secondary alterations induced by apoptosis-associated caspase action, irregular nuclear margins and focal diminutions of signal intensity were seen by immunohistochemistry in FTLD-MAPT-P301L carriers. Cytoplasmic lamin B1 puncta in human brains (Fig. 8, Table 1) may reflect apoptotic bodies from partitioning of cellular contents. Conversely, nuclear clefts detected with lamin B1 antibody were also present in other neurodegenerative diseases; they likely reflect age-dependent changes but could nonetheless comprise a comorbidity to exacerbate toxic effects of tau accumulation. Overall, the propensity of tau conformer to condense on the nuclear envelope could be the important pathogenic mechanism at the cellular level with this hypothesis being supported by other analyses of tauopathy [14, 19] and with parallel effects considered for other neurodegenerative diseases [8–13].

### Condensed tau as a toxic intermediator

The decline in cell viability following tau seeding activity mirrors previous observations for certain types of tau seeds [39, 40]. Concerning the pathways decreasing cell viability, soluble oligomeric, but not monomeric nor fibrillar, forms of tau have been considered to be cytotoxic due to their ability to be internalized into recipient cells and recruit monomeric tau into filamentous inclusions [64–70]. More recently, several lines of evidence suggest that tau uptake and aggregation are not sufficient per se to cause immediate neuronal cell death [71, 72]. Moreover, the presence of neurofibrillary tangles (NFTs) does not inevitably lead to neuronal and network dysfunction in vivo [73]. Experiments described here show that a liquid state condensed tau resulting from the introduction of pathogenic seeds containing the tau conformer ensemble typed with a chemical denaturation profile CSA Type 2, as noted previously, a signature associated with a diagnosis of svPPA [6], is recruited to the nuclear envelope and triggers disruption of nuclear-cytoplasmic transport. In turn, the response of compromised cells is to initiate a regulated cell death process, this pathway having the hallmarks of apoptosis in the HEK-derived ES1 cells, as shown by the production of cleaved caspase 3, accumulation of Bax dimers, and microscopic observation of apoptotic bodies (Fig. 4). Taken together, our findings imply a potentially important toxic intermediate which is a demixed liquid state polymeric tau which could accelerate neurologic deterioration with propagation of intracellular tau aggregates.

## Conclusions

Our data support an important pathological mechanism for disease-associated tau that relates to a behavioral variant of frontotemporal dementia, especially those with a tau conformational profile designated as CSA Type 2. Specifically, the cloned pathogenic tau conformers associate with the nuclear envelope and give rise to condensed phase forms that initiate regulated cell death by hindering nuclear-cytoplasmic transport. While interactions with other cellular milieu biopolymers that affect LLPS remain of interest, once tau LLPS occurs (as prompted here by a repeat domain FTLD-MAPT P301L mutation), we infer that these species reduce cell viability in the intermediates stage of disease and may serve to germinate fibrillar forms seen at endpoint [26, 27, 74]. This sequence of pathogenic events indicates that the transition from a “cloud” of conformers in a prodromal state [6] to an ensemble of tau conformers could be a more relevant target for disease intervention.

## Methods

### Brain tissues of patients and transgenic mice and immunohistochemistry

FTLD-MAPT-P301L patients of both sexes were as described previously [54] and as per Table 1. Clinical features of the patients were assessed as per contemporaneous criteria for diagnosis [75, 76]. Control brain samples were obtained from patients who died from non-neurological diseases; diagnostic neuropathology and retrospective chart reviews were carried out for all subjects, with particular attention to ruling out other age-related neurodegenerative diseases as previously described [6]. TgTau^P301L^ mice samples were obtained as described previously [6, 36, 37]. All animal experiments were performed in accordance with local and Canadian Council on Animal Care ethics guidelines.

Brain tissues from patients were processed for histologic and immunohistochemical purposes as described previously [6]. Briefly, each specimen was fixed in neutral buffered 10% formalin and paraffin-embedded. Six-micrometer sagittal sections were rehydrated and endogenous peroxidase activity was blocked by treatment with 3% hydrogen peroxide for 6 min. The sections were then incubated with primary antibodies at 4 °C overnight: anti-phospho-tau mAb, AT8 (1:200, MN1020, Thermo Fisher); anti-Lamin B1 pAb (1:200, ab16048, Abcam). The target molecules were visualized with horseradish peroxidase using the DAKO ARK kit according to the manufacturer’s instructions. Nuclei were counterstained using Mayer’s hematoxylin or hoechst 33342 (Invitrogen, H1399), dehydrated, and cover-slipped with permanent mounting medium. The section images were acquired with NanoZoomer 2.0-RS digital slide scanner (Hamamatsu) and analyzed using NDP.view2 (Hamamatsu) and Image J software (https://imagej.nih.gov/ij/). Assessment of lamin B immunohistochemistry in the dentate gyrus of FTLD-MAPT-P301L cases and controls (Table 1) was performed in a semiquantitative way by two observers at a multiheaded microscope at × 40 magnification. Complete coverage of the dentate gyrus within the medial-posterior hippocampus was obtained by observing 20–30 fields at this magnification, using an analogous coronal section from subjects sampled at the level or immediately rostral to the lateral geniculate. For clefts, cytoplasmic staining and discontinuous staining intensity: 0.5 = rare, 1–5 neurons affected in one field; 1 = mild, 1–5 neurons affected per field in more than one field; 2 = moderate, 6–15 neurons per field at least in one field (usually several): moderate; 3 = severe, > 15 neurons affected per field at least in one field (usually several). A similar scheme was used for angular nuclear margins: 0.5 = rare, 1–5 neurons affected in one field; 1 = mild, 1–5 neurons affected per field in more than one field; 2 = moderate, 6–15 neurons per field or > 15 affected per field but little angled; 3 = severe, > 15 neurons affected per field and highly angulated. Tau pathologies were scored as described previously [6, 37].

### Cell culture

To generate doxycycline-inducible GFP-0N4R tau reporter line, an enhanced GFP and human WT 0N4R tau sequences were inserted between the *Bam*HI and *Xho*I restriction sites on the pcDNA5/FRT/TO plasmid (Invitrogen). A short linker sequence (ATCGATGCA) was incorporated between the eGFP coding sequence (CDS) and 0N4R tau CDS within the construct. Site-directed mutagenesis was performed on the resulting plasmid to generate the P301L mutation in the tau CDS (pcDNA5/FRT/TO/GFP-0N4R P301L). The final plasmid and the Flp recombinase vector (pOG44 plasmid, Invitrogen) were packaged with Lipofectamine2000 (Thermo Fisher) and transfected into the Flp-In T-Rex-293 cell line (Invitrogen) according to the manufacturer guidelines. Hygromycin B (Thermo Fisher) was used to select stable integrants that were propagated to generate the final cell line (Dox:GFP-0N4R P301L). To induce expression of GFP-0N4R P301L, doxycycline was added to the culture media at a final concentration of 10 μg/mL. A monoclonal HEK293 cell line stably expressing a domain of tau with 4 microtubule-binding domain (4RD) with aggregation prone mutations (P301L/V377M) fused to YFP (4RD-YFP P301L/V377M, also known as “Clone 1” and “DS1”) [39, 40] was maintained at 37 °C with 5% CO_2_ in the culture media; Dulbecco’s modified Eagle’s medium (DMEM, 11995-065, Gibco) with high glucose (4.5 g/L) and 2 mM glutamine (Gibco), supplemented with 10% fetal bovine serum (FBS, HyClone) and Penicillin (10 units/mL)-Streptomycin (10 μg/mL) (Gibco). Cells were incubated with cycloheximide (Sigma) at 20 μg/mL for 16 h to suppress translational rate.

### Tau cell seeding assay

The reporter cells were seeded as previously described [6, 37]. Briefly, tau reporter cells were plated at 1 × 10^6^ cells/well of a 12-well culture plates and, on the next day, seeded with liposome-protein complexes derived from S1 fractions [38] of brain homogenates (hemi-brains) obtained from a total of eleven aged TgTau^P301L^ mice and wherein matching hemi-brains were fixed, embedded and then staged by immunocytochemistry with AT8 anti-phospho-tau antibody (“Classes I-IV” [37];). To establish the ES1 cell line, the S1 fraction of a TgTau^P301L^ mouse brain that predominantly produced the nuclear envelope-associated tau inclusions (69.5 ± 12.1% SEM, *n* = 5 independent fields) was used for seeding. Two microliters of brain homogenate (5–8 mg/mL protein solution was adjusted by total tau content to 8 μg/mL based on the estimation of conformation-dependent immunoassay) [6] were combined with the same volume of Lipofectamine 3000 (L3000-015, Thermo Fisher Scientific) and added to the wells. The cells were then incubated for 6 h at 37 °C and the media containing the liposome-protein complex were replaced with fresh culture media.

### Single-cell cloning by limiting dilution

The cells were resuspended and counted using the automated cell counter, Countess (Invitrogen) (see “Cell viability”). Two hundred microliters of the cell suspensions with concentration of 3 cells/mL were added to each well of 96-well culture plates. Single-cell clones in each well were inspected after 4 days and then at two-day intervals. The cell clones verified as originating from one center of growth were subcultured and frozen in liquid nitrogen until use.

### Cell viability

Cells were resuspended by trypsinization and stained with the same volume of trypan blue (Invitrogen). The samples were loaded into the chamber ports on one side of the Countess cell counting chamber slide (Invitrogen). Viable and dead cells were counted using the automated cell counter (“Countess”; Invitrogen) using a trypan blue exclusion assay. Viability was expressed as a percentage of live cells to total cells counted. Cell viability was also determined based on lactate dehydrogenase (LDH) activity in conditioned culture media using a commercial kit (G1780, Promega) following the manufacturer’s instructions. Culture supernatants were collected and incubated with tetrazolium salt, as the substrate, for 30 min at room temperature. The red formazan products of the enzymatic reaction were quantified using a microtiter plate reader (μQuant, Bio-Tek) at a wavelength of 490 nm. The LDH activities were expressed as a percentage to the control conditioned media.

### Immunocytochemistry and live cell imaging

Cells were plated on poly-D-lysine (Sigma) and laminin (Sigma) double-coated microscope cover glasses (Thermo Fisher Scientific). For immunocytochemistry, cells were fixed in paraformaldehyde (4%, pH 7.4, Electron Microscopy Sciences) for 15 min and optionally permeabilized with PBS containing Triton X-100 (0.1%). The fixed cells were blocked with 1% BSA in PBST (PBS with 0.1% Tween 20) for 30 min and probed with mAb or pAb at 4 °C overnight: anti-nuclear pore complex proteins mAb (1:2000, ab24609, NPC414, Abcam) and anti-Lamin B1 pAb (1:2000, ab16048, abcam). To visualize the target molecules, cells were then incubated with Alexa Fluor 594- (1:2000, Invitrogen, A32742) or 660-conjugated secondary antibodies (1:2000, Invitrogen, A-21073). For amyloid fibril staining, cells were incubated with thioflavin S (ThS, 20 μg/mL in PBST) for 15 min and differentiated with 50% ethanol for 10 s at room temperature. Counterstaining for nuclei was performed with hoechst 33342 (Invitrogen). A multi-track configuration with laser excitation (ex) lines and emission (em) filters for confocal microscopy were as follows: YFP, 488 nm ex and 530 nm em; CFP (for thioflavin S), 405 nm ex and 420–475 nm em; hoechst 33342, 405 nm ex and 385–420 nm em; Alexa Fluor 594, 590 nm ex and 617 nm em; Alexa Fluor 660, 663 nm ex and 691 nm em.

Cells were then imaged and analyzed by the laser scanning confocal microscope. Analysis of tau inclusion particles was restricted to those over 500 nm in diameter (corresponding to > 0.2 μm^2^), due to a 250 nm lateral resolution limit commonly encountered for confocal microscopes [77]. Morphometric shape descriptors including circularity, solidary, roundness, and cross-sectional area were measured using ImageJ. Manders’ colocalization coefficients were calculated using Coloc 2 ImageJ plugin (https://imagej.nih.gov/ij/). Signal intensity of tau was determined by Plot Profile analysis using ImageJ software. For live cell imaging, tau reporter cells were cultured on a μ-Dish 35-mm plate (81156, ibidi), seeded with pathogenic tau derived from TgTau^P301L^, and analyzed by live cell imaging. At 6 days post-seeding, time-lapse images of the cells were collected for 12–18 h (10 min/frame for 72–108 frames) with Z-stack function under identical imaging settings. Image data were acquired with the laser scanning confocal microscope, ZEN Digital Imaging for LSM 700 (Zeiss) fitted with an environmental chamber at 37 °C and 5% CO_2_ and analyzed using Zen 2010b SP1 imaging software (Zeiss) and ImageJ (https://imagej.nih.gov/ij/).

### Transmission electron microscopy (TEM)

Cells were collected and fixed in pre-warmed 2% paraformaldehyde in PB (0.1 M phosphate buffer, pH 7.3) for 20 min at 37 °C and another 40 min at room temperature. The samples were post-fixed in 1% osmium tetroxide in PB for 1 h and then incubated with 1% carbohydrazide in distilled water for 10 min at room temperature. After additional incubation with 1% osmium tetroxide for 1 h, the samples were dehydrated in an ethanol series and infiltrated with an increasing concentration of Spurr’s resin (14300, Electron Microscopy Sciences) over several days. The infiltrated cell pellets were transferred to beam capsules and polymerized at 65 °C for 24 h. The resin-embedded pellets were sectioned with a thickness of 100 nm and incubated in 0.5% uranyl acetate for 1 h at RT for negative staining. The thin sections on carbon grids were imaged using JEM-2100 LaB6 TEM (JEOL) with Gatan DigitalMicrograph (Gatan) software operated at 25 kV. TEM images were then analyzed using ImageJ software.

### Nuclear-cytoplasmic compartmentalization (NCC) assay

Cells were transfected with the NCC reporter construct which carries the IRES-linked sequences for GFP fused nuclear export signal (NES) and RFP fused nuclear localization signal (NLS) under the control of EF1α promoter (pLVX-EF1alpha-2xGFP:NES-IRES-2xRFP:NLS) [53]. One microgram of the construct was combined with 2 μL Lipofectamine 3000 (L3000-015, Thermo Fisher Scientific) and added to the cells. The cells were then incubated for 6 h at 37 °C and the media containing the DNA-liposome complex were replaced with fresh culture media. After 24 h, images were obtained using the laser scanning confocal microscope as described above (see “Immunocytochemistry and live cell imaging”).

### Fluorescence recovery after photobleaching (FRAP) analysis

For FRAP analysis of NCC, cells were plated on a μ-Dish 35-mm plate and transfected with NCC reporter construct (see “Nuclear-cytoplasmic compartmentalization (NCC) assay”). On the next day, RFP signals in nuclear region of interests (ROIs) were obtained as time-lapse images (10 min/frame for 5 frames) (see “Immunocytochemistry and live cell imaging”) and then RFP was repeatedly bleached throughout the entire field. To determine the recovery of RFP in nuclear ROIs, post-bleaching time-lapse images were collected for 6 h (10 min/frame for 36 frames). Intensities of RFP in nuclear ROIs were measured using ImageJ software. For FRAP analysis of condensed liquid tau droplets, ES1 cells were plated on a μ-Dish 35 mm plate and reference images were obtained. ROIs including nuclear envelope tau inclusions were repeatedly bleached and time-lapse images were collected for 30 min (30 s/frame for 55 frames).

### Sedimentation analysis

Sedimentation of tau in the seeded reporter cells was performed as previously described [39, 40] with some modifications. Briefly, clarified cell lysates were prepared as described above (see “Limited proteolysis”) and 10% of each lysate was set aside as total fractions. The rest were centrifuged at 100,000×*g* for 1 h, and the supernatants were placed aside as soluble fractions. The pellet was washed with 1.5 mL PBS prior to ultracentrifugation at 100,000×*g* for 30 min. For insoluble fractions, the pellet was resuspended in RIPA buffer (50 mM Tris, 150 mM NaCl, pH 7.4, 1% NP-40, 0.5% sodium deoxycholate, 4% SDS and 100 mM DTT) and sonicated at 30 amplitude for 3 min. Protein concentrations were normalized by BCA protein assay (Pierce) and tau in each fraction were analyzed by the capillary western assay.

### Western blot and capillary western assays

Protein concentrations of each sample were normalized by BCA protein assay (Pierce). The samples were resolved on 15% Tris-Glycine gels or NuPAGE Bis-Tris gels (NP0343, Invitrogen) and transferred to PVDF membrane (Thermo Fisher Scientific). The membranes were blocked with 2% bovine serum albumin (BSA, Darmstadt) in TBST (TBS with 0.1% Tween 20) and probed with monoclonal (mAb) or polyclonal (pAb) antibodies at 4 °C overnight: anti-tau mAb ET3 [78] (1:500); anti-tau mAb RD4 (1:5,000, 05-804, Millipore); anti-Cleaved Caspase-3 pAb (1:2,000, #9661, Cell Signaling Technology); anti-Bax mAb (1:2,000, ab32503, abcam); anti-β-actin mAb (1:10,000, Abcam, ab20272); anti-ribosomal protein S6 (1:1,000, #2217, Cell Signaling Technology); anti-phospho-S6 ribosomal protein (1:1,000, #2215, Cell Signaling Technology). Anti-mouse IgG pAb conjugated to horseradish peroxidase (1:10,000, 170-6516, Bio-Rad) or anti-rabbit IgG pAb conjugated alkaline phosphatase (1:10,000, S3731, Promega) were used as secondary antibodies and visualized by detecting chemiluminescence (32209, Pierce) or fluorescence (S1000, Promega) signals. The membranes were stripped in western blot stripping buffer (46430, Thermo Fisher Scientific) and re-probed as needed.

Capillary western was performed as described in a previous report [79]. Reagents and equipment were purchased from ProteinSimple unless stated otherwise. Cell lysates or fractions were incubated with Fluorescent Master Mix at 95 °C for 5 min. Four microliters of each sample were loaded into the top-row wells of plates preloaded with proprietary electrophoresis buffers designed to separate proteins of 12–230 kDa. Subsequent rows of the plate were filled with blocking buffer, primary and secondary antibody solutions, and chemiluminescence reagents, according to the manufacturer’s instructions. Primary antibodies were anti-tau mAb ET3 [78] (1:50) and anti-β-tubulin pAb (1:1000, NB600-936, Novus Biologicals). Secondary antibodies were anti-mouse or anti-rabbit secondary HRP conjugate. Peak area calculations and generation of artificial lane views were performed by the Compass software using the default Gaussian method.

### Limited proteolysis

Cell pellets were lysed by triturating in PBS containing 0.05% Triton X-100 and protease inhibitors (cOmplete, Roche) and clarified by 5 min sequential centrifugations at 500×*g* and 1000×*g*. The cell lysates (1 μg/μL) were enzymatically digested with 50 μg/mL pronase E (Roche) at 37 °C for 1 h followed quenching with protease inhibitors and SDS-PAGE loading buffer, 15 μg/mL proteinase K (Ambion) at 37 °C for 1 h followed quenching with SDS-PAGE loading buffer, and 40 μg/mL thermolysin (Sigma) at 65 °C for 30 min followed quenching with 0.5 M EDTA and SDS-PAGE loading buffer, respectively. The undigested tau fragments in each enzymatic reaction were determined by western blot analysis using anti-tau mAb ET3 [78] or anti-tau mAb RD4 (Millipore). For details, see western blot and capillary western assays above.

### Statistical analysis

The number of biological and technical replicates of compared groups were at least *n* = 3 for each observation and represented in corresponding figure legends. Sample size (*n*) indicates biological replicates unless otherwise stated. For the most statistical analysis, comparisons of means were performed using the unpaired, two-tailed Student *t* test. The fluorescence recovery of individual nuclei in the FRAP analysis (Fig. 7d) and the assessment of nuclear deformation (Fig. 8b) were compared using ANOVA with post hoc Tukey’s multiple comparison test. The circularity of YFP and ThS double-positive tau inclusions (Fig. 3f) and the western blot analysis of translational efficiency of the ES1 cells (Fig. 4g) were also statistically compared to each other using ANOVA with Tukey’s post hoc analysis. The equality of variance in the nuclear shape descriptors was analyzed by F-statistics (Fig. 5b, Supplementary Figure 6b). Statistical analysis of all data was performed using PRISM version 5 software (GraphPad Software).

## Supplementary Information

**Additional file 1: Supplementary Figure 1.** Tau inclusions transfer through tunneling nanotube-like membrane extension. The 4RD-YFP tau reporter cells were seeded as per Fig. 1. **a.** Tau inclusions transferred between cells; those separated approximately 20 μm from each other (middle) or clumped together (bottom), were investigated using time-lapse imaging for 12 hours (10 min/frame for 72 frames). **b.** The cells connected with others by way of multiple tunneling nanotube-like membrane extension. Arrowheads point to cell-to-cell movements of tau inclusion. Scale bars, 10 μm.

**Additional file 2: Supplementary Figure 2.** Droplet-like behavior of tau inclusion in the seeded reporter cells. **a.** Heterogeneous morphologies of tau inclusions obtained by transduction of seed-competent pathogenic tau into the 4RD-YFP tau reporter cells as per Fig. 1, including amorphous large tau inclusions (amorphous), discontinuous perimeter signals along with the nuclear edges (nuclear envelope), and small bead shapes with various sizes most likely seen in the nucleus (speckle). Scale bars, 10 μm. **b.** Droplet fusions in doxycycline-inducible GFP-0N4R (top) and 4RD-YFP tau reporter cell lines (bottom) seeded as per Fig. 1 were monitored by time-lapse imaging for 16 and 12 hours (10 min/frame), respectively. **c.** Live cell imaging analysis of the seeded 4RD-YFP tau reporter cells as per Fig. 1 revealed that droplet fusions increased the number and cross-sectional area of droplets. The number and total area (μm^2^) were measured every 3 hours. At the bottom left corner, nuclear envelope tau signals appeared approximately 6 to 9 hours after the transduction. Scale bar, 10 μm.

**Additional file 3: Supplementary Figure 3.** Thioflavin S staining for intracellular tau aggregates in ES1 cells. ES1 cells (**a**) and control reporter cells (**b**) were fixed and incubated with thioflavin S (ThS, 20 μg/mL). The images were acquired using cyan fluorescent protein (CFP for ThS) and enhanced yellow fluorescent protein channels (YFP for tau). Cell images were from 8 and 4 different areas for ES1 and control, respectively (160 μm^2^ for each). Tau in green; ThS in blue. Scale bar, 20 μm.

**Additional file 4: Supplementary Figure 4.** Protease-resistant core of the aggregated tau in ES1 cells. ES1 cells were re-subcloned by limiting dilution to obtain sublines. To differentiate the protected fibrillar cores of tau aggregates in the individual cells, the cell lysates (**a**) were digested using pronase E (**b**), proteinase K (**c**), and thermolysin (**d**), and analyzed by western blot using anti-tau antibodies, ET3 or RD4. The limited proteolytic digestions revealed resistant core peptides in each subline (1 to 6) ranging from 10 to 25 kDa in size, while tau species in the reporter controls (Ctrl, 4RD-YFP) were completely cleaved. The 10 kDa protease-resistant core appeared in all digestion conditions, and one or two bands between 15 to 20 kDa were shown depending on the enzymes tested. The patterns of the fragmented resistant cores were identical to each other.

**Additional file 5: Supplementary Figure 5.** Lamin B1 staining for morphometric analysis of nuclei in ES1 cells. ES1 line (**a**) and control reporter cells (**b**) were fixed and probed with anti-lamin B1 antibody. The images were acquired from 4 different areas (160 μm^2^ for each), respectively. The nuclear margins were selected along the lamin B1 stain (as region of interest, ROI) and used to measure morphometric shape descriptors, including solidity, circularity, roundness, and area. ROIs in red; tau in green; lamin B1 in magenta; nuclei in blue. Scale bar, 20 μm.

**Additional file 6: Supplementary Figure 6.** Nuclear deformation in ES1 cells. Panels **a** and **b.** Nuclei in the control reporter and ES1 cells were visualized using lamin B1 staining as per Fig. 5a. **a.** Specificity of the lamin B1 immunoreactivity was confirmed in the absence and presence of the primary antibody. Tau in green; lamin B1 in magenta; nuclei in blue. Scale bar, 10 μm. **b.** The roundness and size (cross-sectional area, μm^2^) of individual nuclei were measured along the nuclear margins. *n* = 325 and 245 for control and ES1 cells, respectively. ***p < 0.001 in comparison with the controls. **c.** TEM analysis of ES1 cells as per Fig. 5d and e. Nuclear ruptures were indicated by red arrowheads. C, cytoplasm; N, nucleoplasm. Scale bar, 500 nm and 250 nm in the boxed images.

**Additional file 7: Supplementary Figure 7.** Nuclear-cytoplasmic compartmentalization in ES1 cells. Control reporter cells (**a**) and ES1 cells (**b**) were transiently transfected with nuclear-cytoplasmic compartmentalization (NCC) reporter construct and imaged at 24 hours post transfection. The images were obtained from 8 different areas (160 μm^2^ for each), respectively. Faint dispersed (in the control) and strong aggregated tau signals (in ES1) were often overlapped with green fluorescent protein (GFP) signals derived from the NCC reporter. RFP, red fluorescent protein (in magenta). Scale bar, 20 μm.

**Additional file 8: Supplementary Movie 1.** Description: Time-lapse imaging of GFP-0N4R reporter cells seeded with S1 brain fractions including no seed-competent tau derived from aged non-transgenic mice. Images were obtained for 12 hours 30 min (10 min/frame for 75 frames). Scale bars, 10 μm.

**Additional file 9: Supplementary Movie 2.** Description: Time-lapse imaging of GFP-0N4R reporter cells seeded with S1 brain fractions including pathogenic tau derived from aged TgTau^P301L^ mice with neurological signs. Images were obtained for 12 hours 30 min (10 min/frame for 75 frames). Scale bars, 10 μm.

**Additional file 10: Supplementary Movie 3.** Description: Time-lapse imaging of 4RD-YFP reporter cells seeded with S1 brain fractions including no seed-competent tau derived from aged non-transgenic mice. Images were obtained for 5 hours (5 min/frame for 60 frames). Scale bars, 10 μm.

**Additional file 11: Supplementary Movie 4.** Description: Time-lapse imaging of 4RD-YFP reporter cells seeded with S1 brain fractions including pathogenic tau derived from aged TgTau^P301L^ mice with neurological signs. Tau transfer through tunneling nanotube-like membrane extension on a bottom focal plane (Z4). Images were obtained for 5 hours (5 min/frame for 60 frames). Scale bars, 10 μm.

**Additional file 12: Supplementary Movie 5.** Description: Time-lapse imaging of the seeded 4RD-YFP reporter cells shown in Supplementary Movie 4. Tau transfer through tunneling nanotube-like membrane extension on a middle focal plane (Z7). Images were obtained for 5 hours (5 min/frame for 60 frames). Scale bars, 10 μm.

**Additional file 13: Supplementary Movie 6.** Description: Time-lapse imaging of 4RD-YFP reporter cells seeded with S1 brain fractions including no seed-competent tau derived from aged non-transgenic mice. Images were obtained for 12 hours 30 min (10 min/frame for 75 frames). Scale bars, 10 μm.

**Additional file 14: Supplementary Movie 7.** Description: Time-lapse imaging of 4RD-YFP reporter cells seeded with S1 brain fractions including pathogenic tau derived from aged TgTau^P301L^ mice with neurological signs. Images were obtained for 12 hours 30 min (10 min/frame for 75 frames). Scale bars, 10 μm.

**Additional file 15: Supplementary Movie 8.** Description: Time-lapse imaging of 4RD-YFP reporter cells seeded with S1 brain fractions including pathogenic tau derived from aged TgTau^P301L^ mice with neurological signs. Images were obtained for 7 hours (10 min/frame for 42 frames). Scale bars, 10 μm.

**Additional file 16: Supplementary Movie 9.** Description: Time-lapse imaging of 4RD-YFP reporter cells seeded with S1 brain fractions including pathogenic tau derived from aged TgTau^P301L^ mice with neurological signs. Images were obtained for 7 hours (10 min/frame for 42 frames). Scale bars, 10 μm.

**Additional file 17: Supplementary Movie 10.** Description: FRAP analysis of nuclear envelope tau inclusions and fusion of droplet-like inclusions in ES1 cells. For FRAP, five reference images were taken at the beginning and nuclear envelope tau inclusions were photobleached. Images were obtained for 30 min at one frame every 30 sec (1/30 frame/sec). Scale bar, 10 μm.

**Additional file 18: Supplementary Movie 11.** Description: Time-lapse imaging of 4RD-YFP reporter cells seeded with S1 brain fractions including pathogenic tau derived from aged TgTau^P301L^ mice with neurological signs. Images were obtained for 13 hours 20 min (10 min/frame for 80 frames). Scale bars, 10 μm.

**Additional file 19: Supplementary Movie 12.** Description: Time-lapse imaging of GFP-0N4R reporter cells seeded with S1 brain fractions including pathogenic tau derived from aged TgTau^P301L^ mice with neurological signs. Images were obtained for 16 hours (10 min/frame for 96 frames). Scale bars, 10 μm.

**Additional file 20: Supplementary Movie 13.** Description: Time-lapse imaging of 4RD-YFP reporter cells seeded with S1 brain fractions including pathogenic tau derived from aged TgTau^P301L^ mice with neurological signs. Images were obtained for 8 hours (10 min/frame for 48 frames). Scale bars, 10 μm.

**Additional file 21: Supplementary Movie 14.** Description: Time-lapse imaging of 4RD-YFP reporter cells seeded with S1 brain fractions including pathogenic tau derived from aged TgTau^P301L^ mice with neurological signs. Images were obtained for 13 hours (10 min/frame for 78 frames). Scale bars, 10 μm.

**Additional file 22: Supplementary Movie 15.** Description: Time-lapse imaging of 4RD-YFP reporter cells seeded with S1 brain fractions including pathogenic tau derived from aged TgTau^P301L^ mice with neurological signs. The cells with nuclear envelope and juxtanuclear tau inclusions underwent apoptotic cell death. Images were obtained for 15 hours 10 min (10 min/frame for 91 frames). Scale bars, 10 μm.

**Additional file 23: Supplementary Movie 16.** Description: Time-lapse imaging of 4RD-YFP reporter cells transiently transfected with NCC reporter construct. For FRAP, five reference images were taken at the beginning and RFP signals were photobleached. Images were obtained for 6 hours 30 min at one frame every 10 min (1/10 frame/min). Scale bar, 10 μm.

**Additional file 24: Supplementary Movie 17.** Description: Time-lapse imaging of ES1 cells transiently transfected with NCC reporter construct. For FRAP, five reference images were taken at the beginning and RFP signals were photobleached. Images were obtained for 6 hours 30 min at one frame every 10 min (1/10 frame/min). Scale bar, 10 μm.

## Data Availability

The datasets used and/or analyzed during the current study are available from the corresponding author on reasonable request.

## Abbreviations

CSA: Conformational stability assay
CFP: Cyan fluorescent protein
FRAP: Fluorescence recovery after photobleaching
FTLD: Frontotemporal lobar degeneration
FTLD-MAPT: FTLD associated with *MAPT* mutations
FTLD-tau: FTLD with tau immunoreactive inclusions
GdnHCl: Guanidine hydrochloride
GFP: Green fluorescent protein
LLPS: Liquid-liquid phase separation
NCC: Nuclear-cytoplasmic compartmentalization
NES: Nuclear export signal
NLS: Puclear localization signal
RFP: Red fluorescent protein
svPPA: Semantic variant of Primary Progressive Aphasia
ThS: Thioflavin S
YFP: Yellow fluorescent protein
