# Supplementary figures and images for: Pathologic tau conformer ensembles induce dynamic, liquid-liquid phase separation events at the nuclear envelope
Source: BMC Biol. 2021 Sep 9;19:199. doi: 10.1186/s12915-021-01132-y (PMC8428099; doi:10.1186/s12915-021-01132-y)

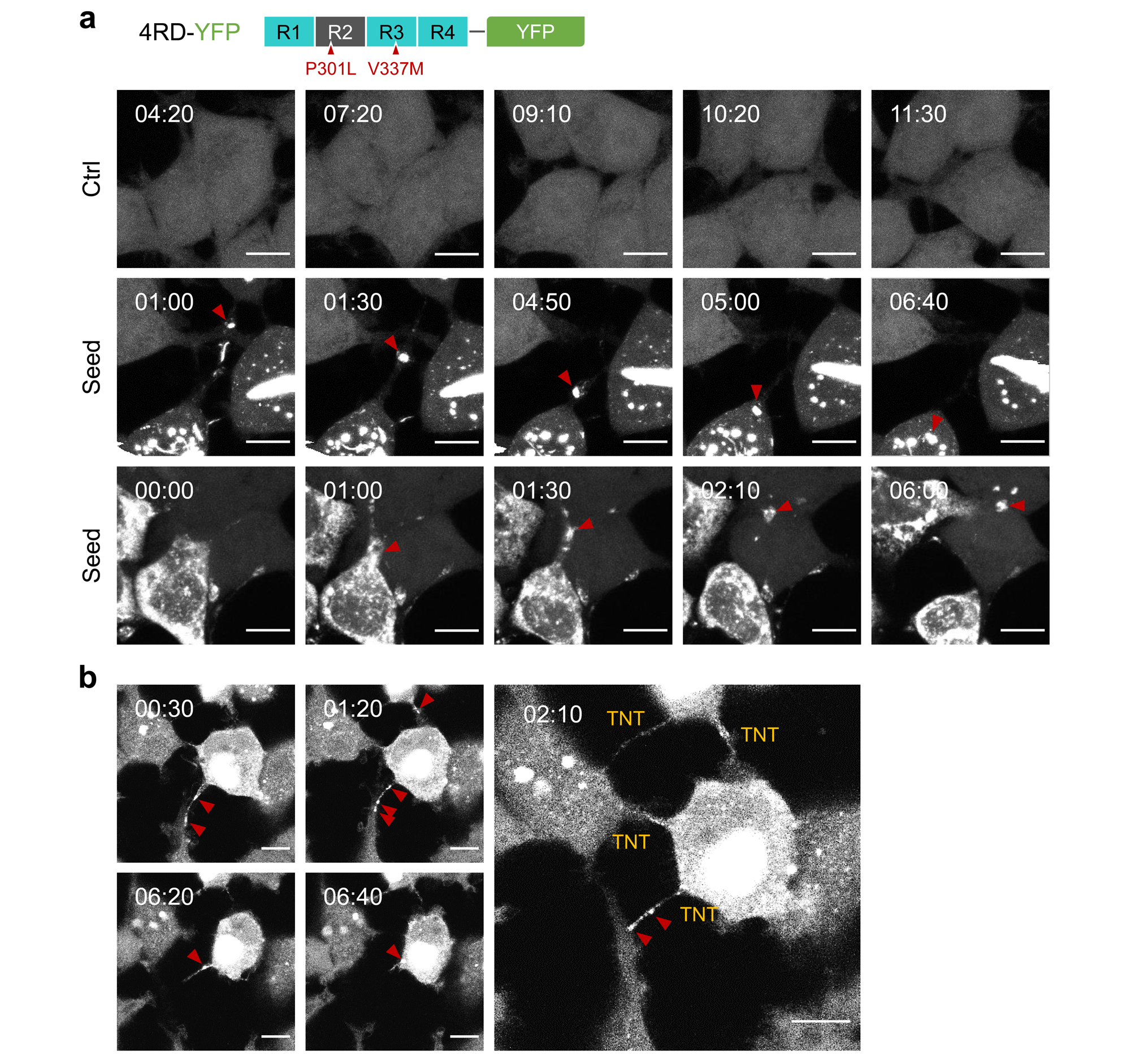

Supplement: Supplementary file 1 — Additional file 1: Supplementary Figure 1. Tau inclusions transfer through tunneling nanotube-like membrane extension. The 4RD-YFP tau reporter cells were seeded as per Fig. 1. a. Tau inclusions transferred between cells; those separated approximately 20 μm from each other (middle) or clumped together (bottom), were investigated using time-lapse imaging for 12 hours (10 min/frame for 72 frames). b. The cells connected with others by way of multiple tunneling nanotube-like membrane extension. Arrowheads point to cell-to-cell movements of tau inclusion. Scale bars, 10 μm. [file 12915_2021_1132_MOESM1_ESM.png]

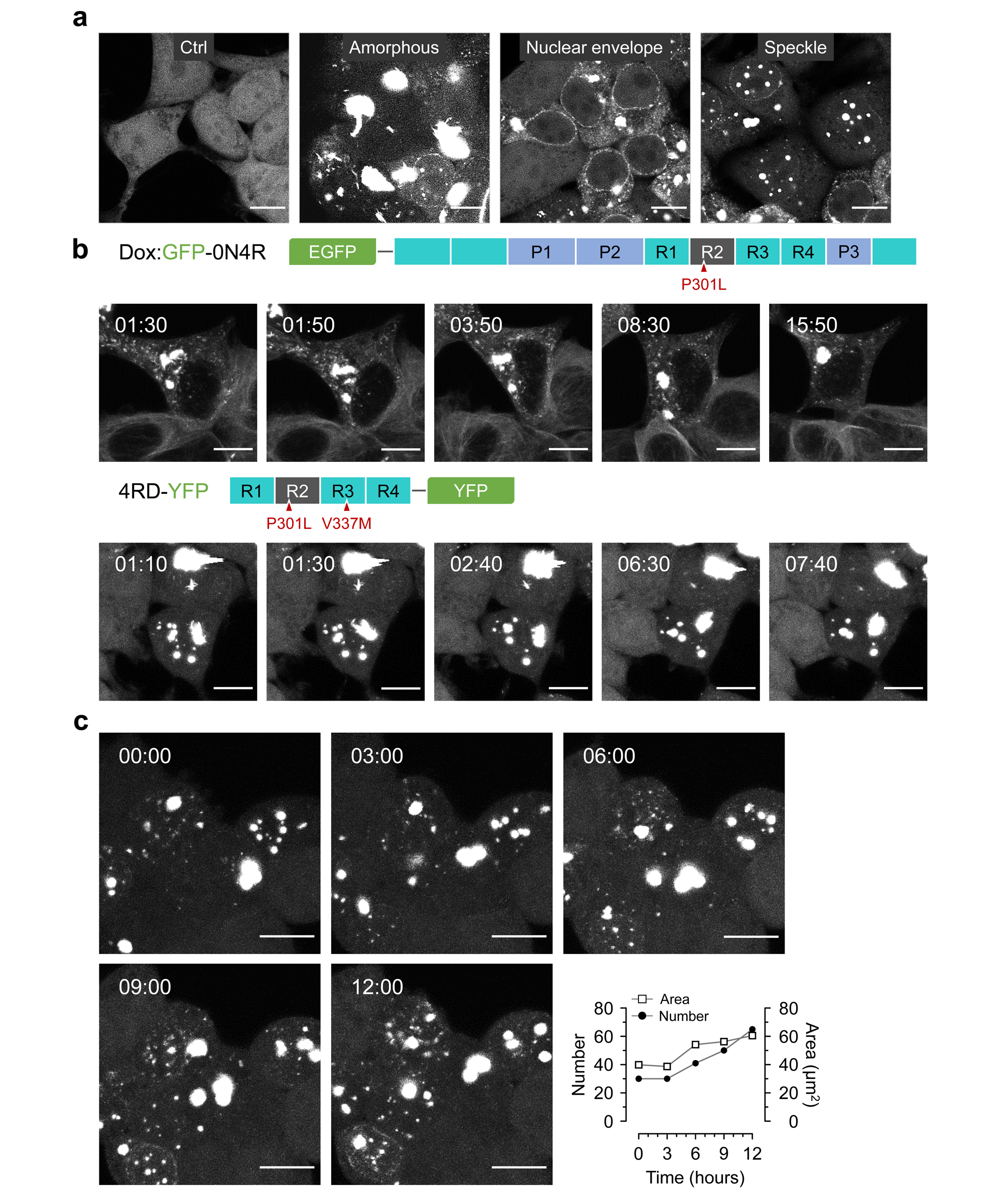

Supplement: Supplementary file 2 — Additional file 2: Supplementary Figure 2. Droplet-like behavior of tau inclusion in the seeded reporter cells. a. Heterogeneous morphologies of tau inclusions obtained by transduction of seed-competent pathogenic tau into the 4RD-YFP tau reporter cells as per Fig. 1, including amorphous large tau inclusions (amorphous), discontinuous perimeter signals along with the nuclear edges (nuclear envelope), and small bead shapes with various sizes most likely seen in the nucleus (speckle). Scale bars, 10 μm. b. Droplet fusions in doxycycline-inducible GFP-0N4R (top) and 4RD-YFP tau reporter cell lines (bottom) seeded as per Fig. 1 were monitored by time-lapse imaging for 16 and 12 hours (10 min/frame), respectively. c. Live cell imaging analysis of the seeded 4RD-YFP tau reporter cells as per Fig. 1 revealed that droplet fusions increased the number and cross-sectional area of droplets. The number and total area (μm2) were measured every 3 hours. At the bottom left corner, nuclear envelope tau signals appeared approximately 6 to 9 hours after the transduction. Scale bar, 10 μm. [file 12915_2021_1132_MOESM2_ESM.png]

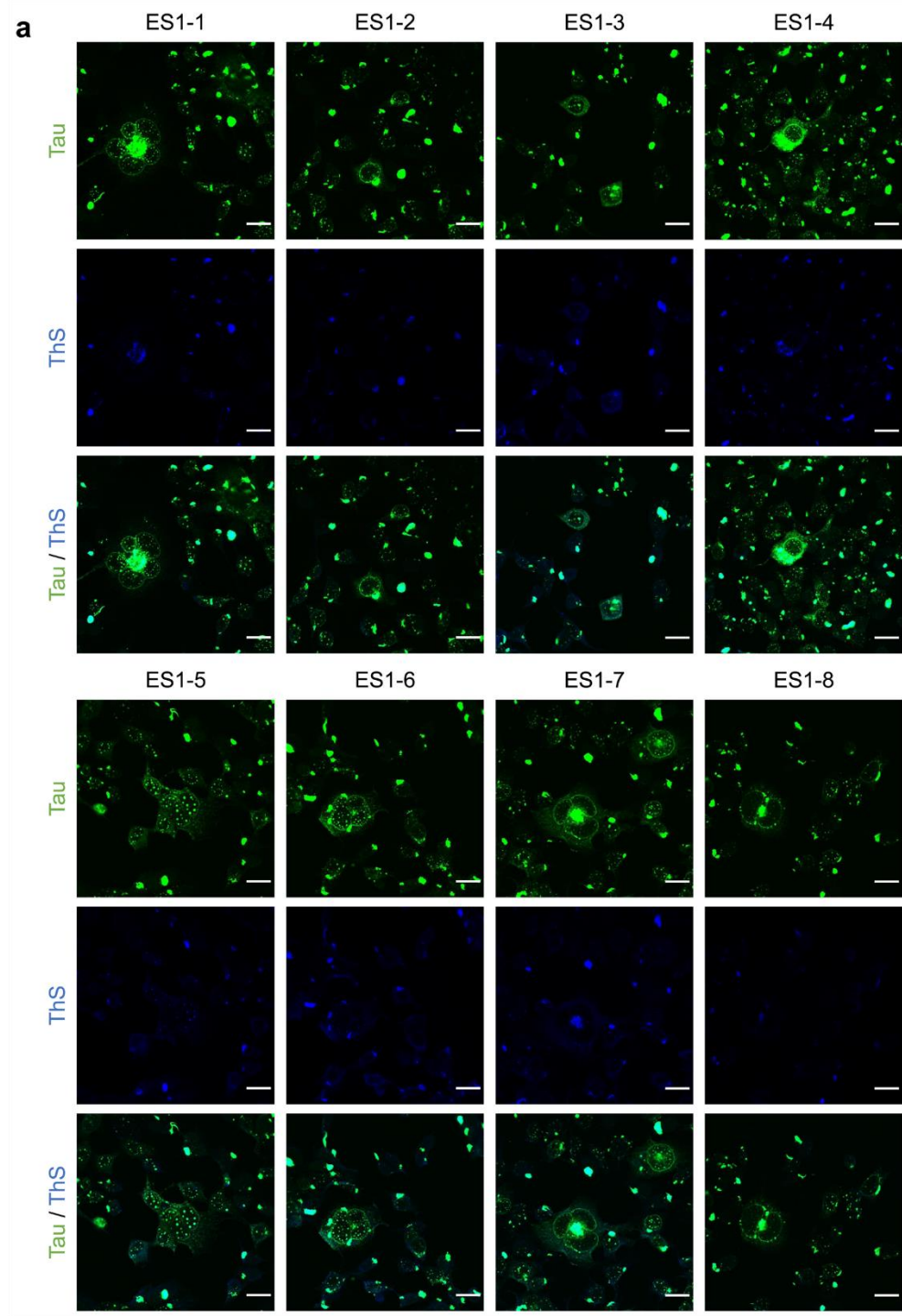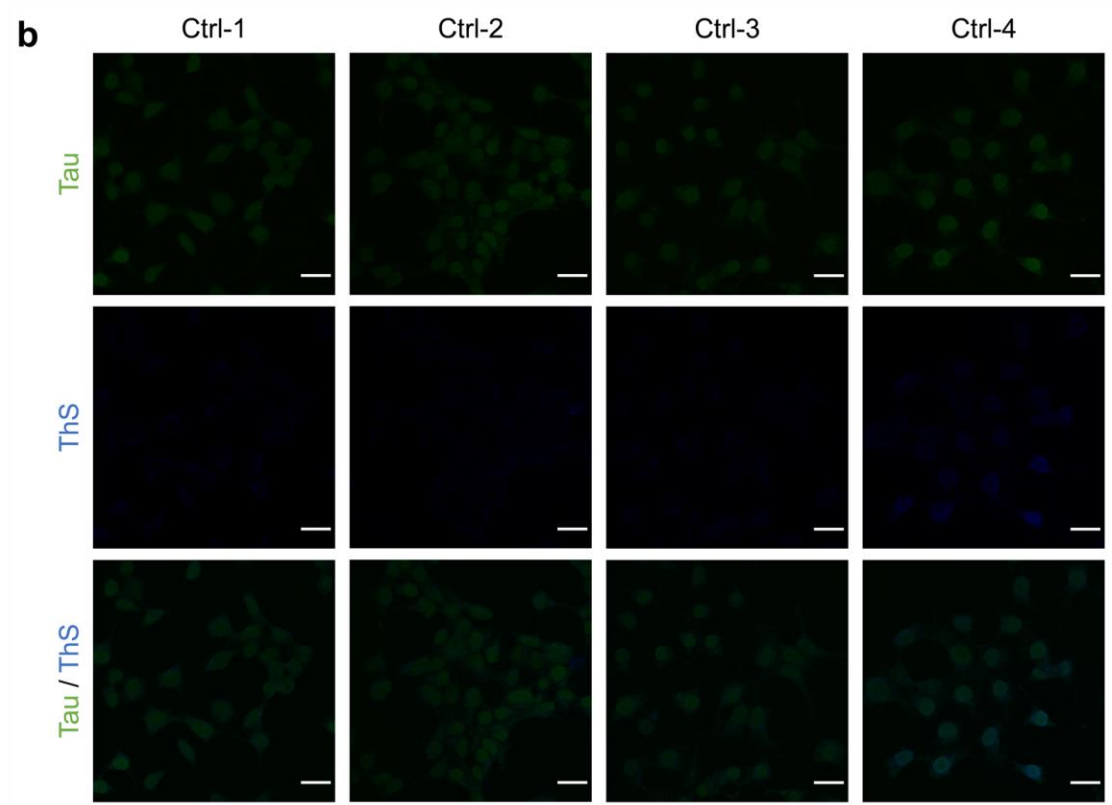

Supplement: Supplementary file 3 — Additional file 3: Supplementary Figure 3. Thioflavin S staining for intracellular tau aggregates in ES1 cells. ES1 cells (a) and control reporter cells (b) were fixed and incubated with thioflavin S (ThS, 20 μg/mL). The images were acquired using cyan fluorescent protein (CFP for ThS) and enhanced yellow fluorescent protein channels (YFP for tau). Cell images were from 8 and 4 different areas for ES1 and control, respectively (160 μm2 for each). Tau in green; ThS in blue. Scale bar, 20 μm. [file 12915_2021_1132_MOESM3_ESM.pdf]

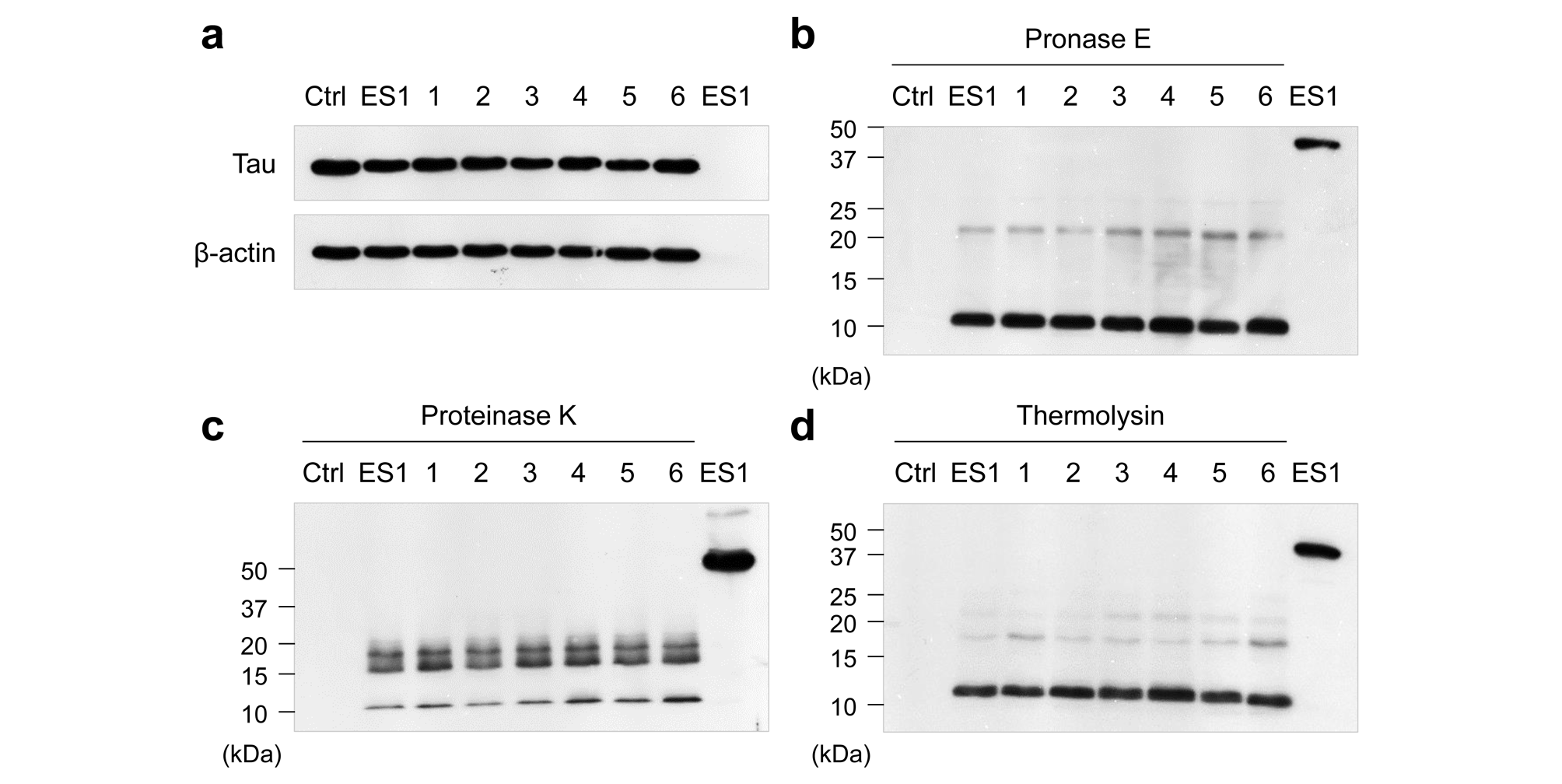

Supplement: Supplementary file 4 — Additional file 4: Supplementary Figure 4. Protease-resistant core of the aggregated tau in ES1 cells. ES1 cells were re-subcloned by limiting dilution to obtain sublines. To differentiate the protected fibrillar cores of tau aggregates in the individual cells, the cell lysates (a) were digested using pronase E (b), proteinase K (c), and thermolysin (d), and analyzed by western blot using anti-tau antibodies, ET3 or RD4. The limited proteolytic digestions revealed resistant core peptides in each subline (1 to 6) ranging from 10 to 25 kDa in size, while tau species in the reporter controls (Ctrl, 4RD-YFP) were completely cleaved. The 10 kDa protease-resistant core appeared in all digestion conditions, and one or two bands between 15 to 20 kDa were shown depending on the enzymes tested. The patterns of the fragmented resistant cores were identical to each other. [file 12915_2021_1132_MOESM4_ESM.png]

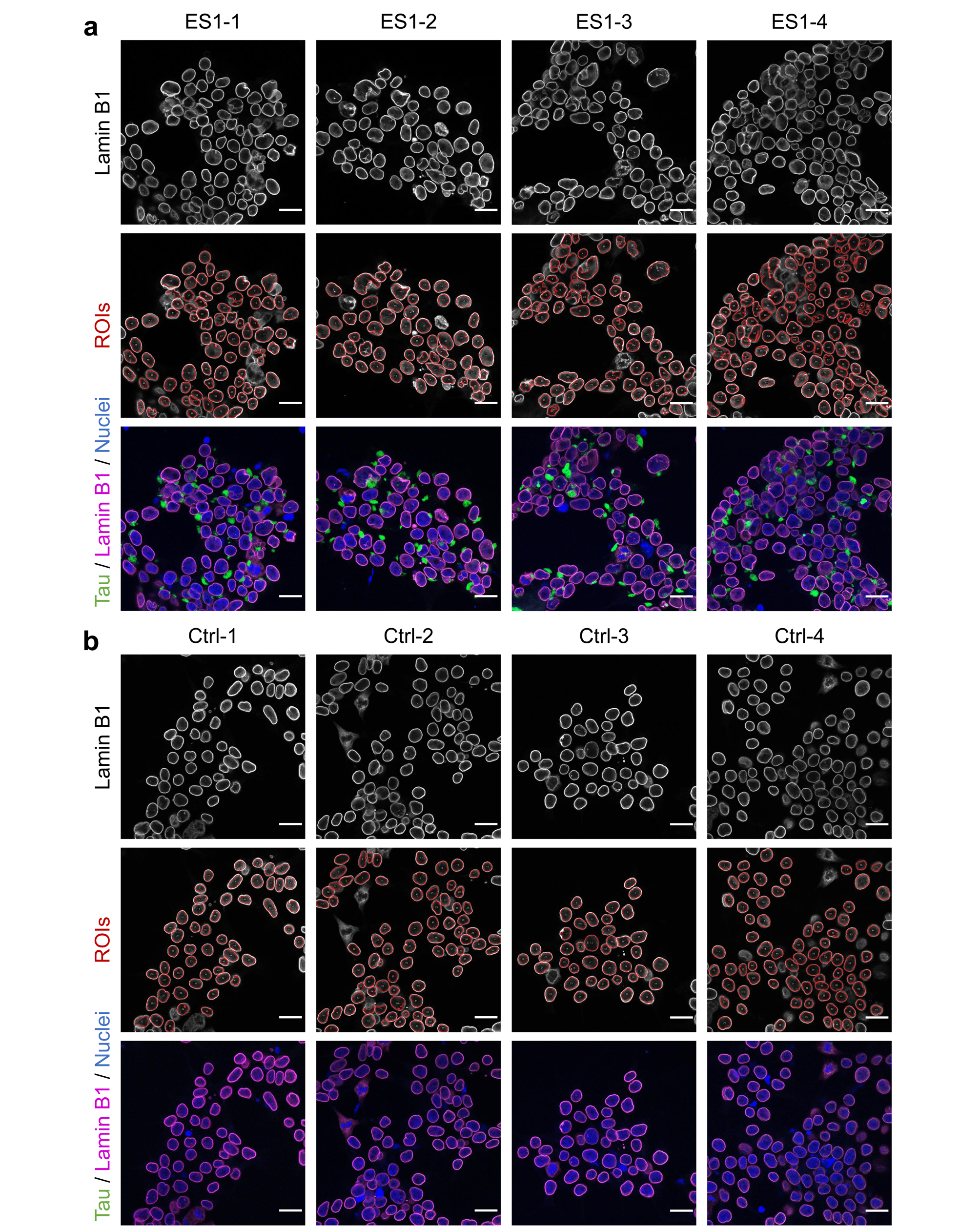

Supplement: Supplementary file 5 — Additional file 5: Supplementary Figure 5. Lamin B1 staining for morphometric analysis of nuclei in ES1 cells. ES1 line (a) and control reporter cells (b) were fixed and probed with anti-lamin B1 antibody. The images were acquired from 4 different areas (160 μm2 for each), respectively. The nuclear margins were selected along the lamin B1 stain (as region of interest, ROI) and used to measure morphometric shape descriptors, including solidity, circularity, roundness, and area. ROIs in red; tau in green; lamin B1 in magenta; nuclei in blue. Scale bar, 20 μm. [file 12915_2021_1132_MOESM5_ESM.png]

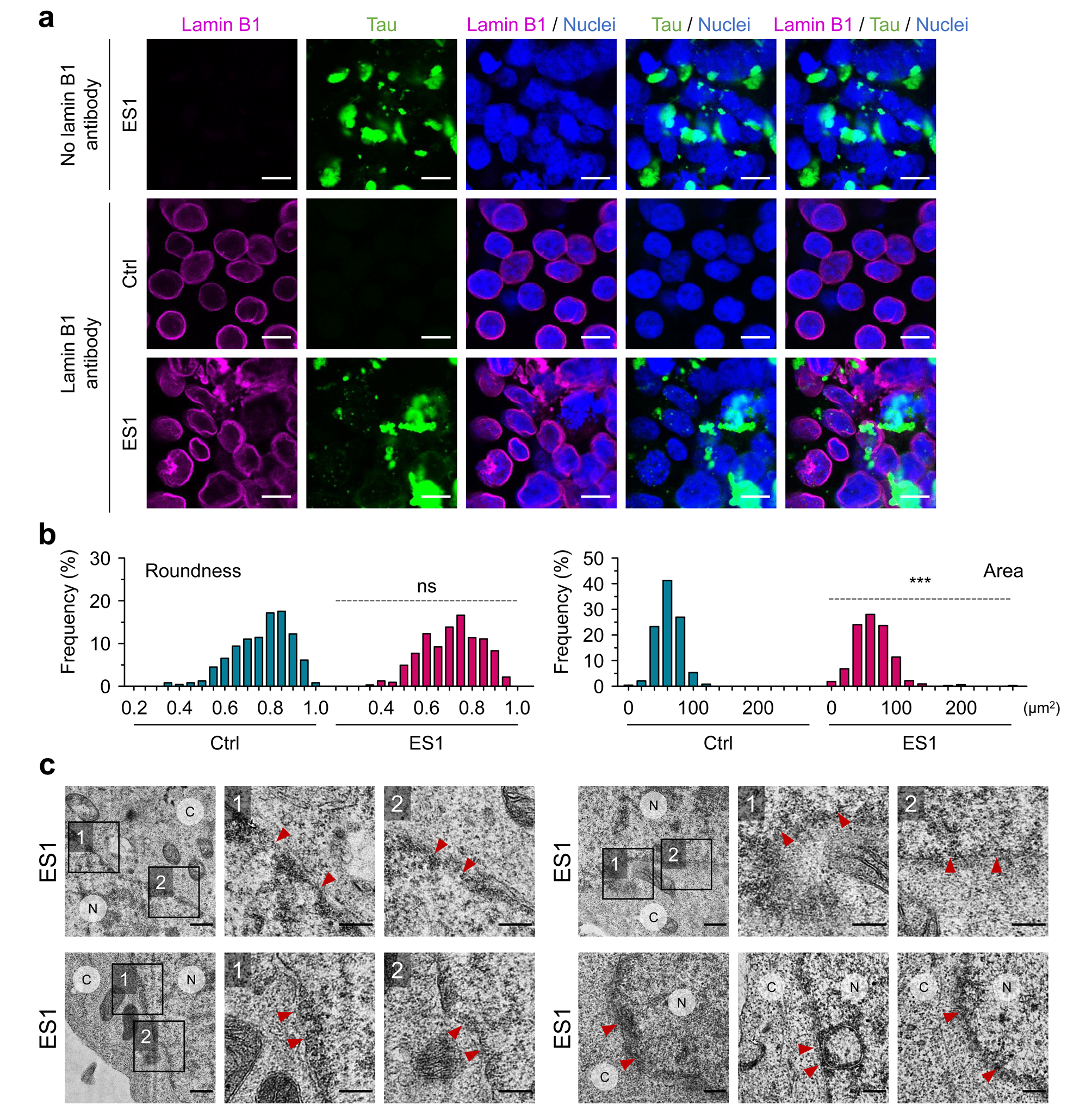

Supplement: Supplementary file 6 — Additional file 6: Supplementary Figure 6. Nuclear deformation in ES1 cells. Panels a and b. Nuclei in the control reporter and ES1 cells were visualized using lamin B1 staining as per Fig. 5a. a. Specificity of the lamin B1 immunoreactivity was confirmed in the absence and presence of the primary antibody. Tau in green; lamin B1 in magenta; nuclei in blue. Scale bar, 10 μm. b. The roundness and size (cross-sectional area, μm2) of individual nuclei were measured along the nuclear margins. n = 325 and 245 for control and ES1 cells, respectively. ***p < 0.001 in comparison with the controls. c. TEM analysis of ES1 cells as per Fig. 5d and e. Nuclear ruptures were indicated by red arrowheads. C, cytoplasm; N, nucleoplasm. Scale bar, 500 nm and 250 nm in the boxed images. [file 12915_2021_1132_MOESM6_ESM.png]

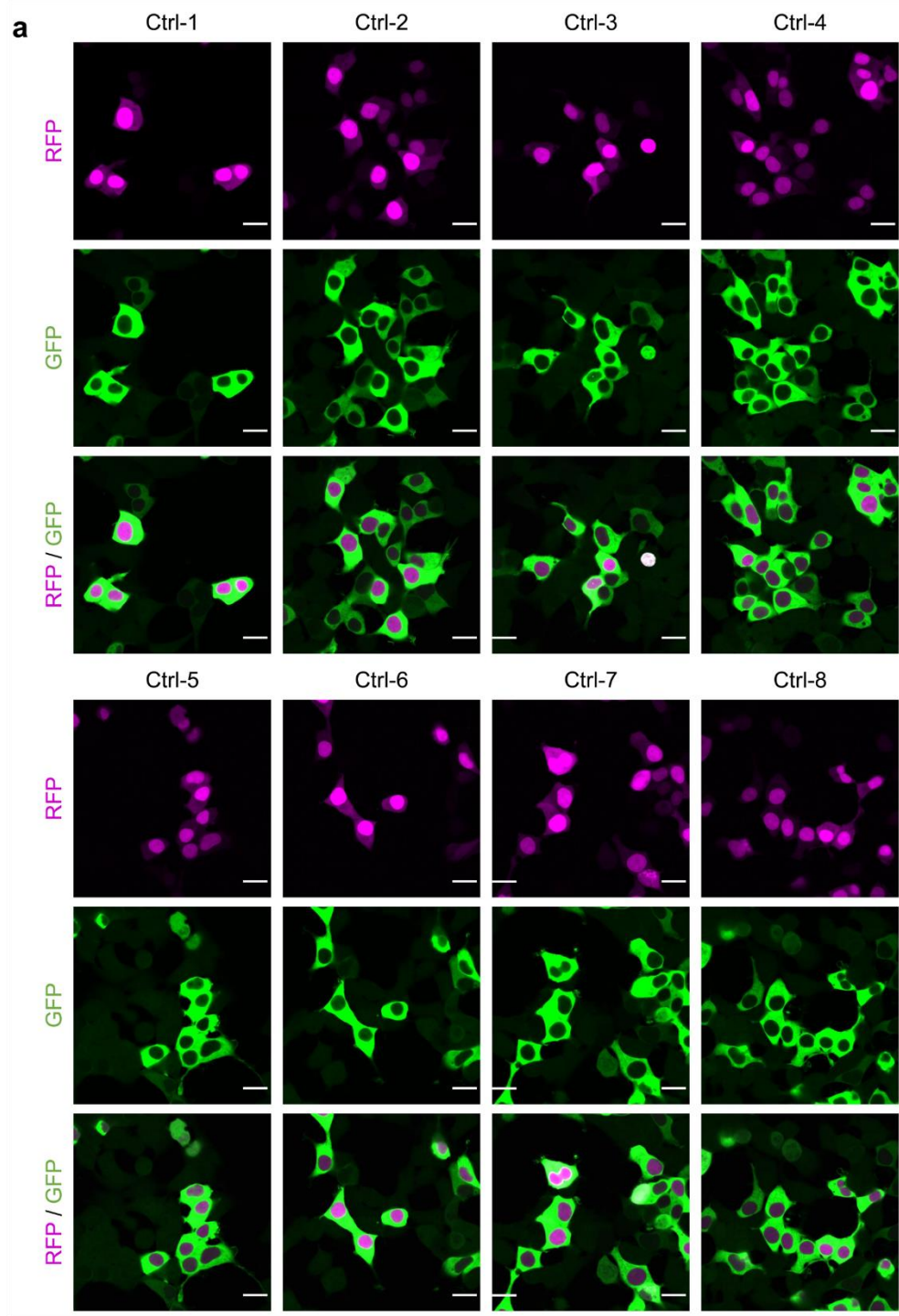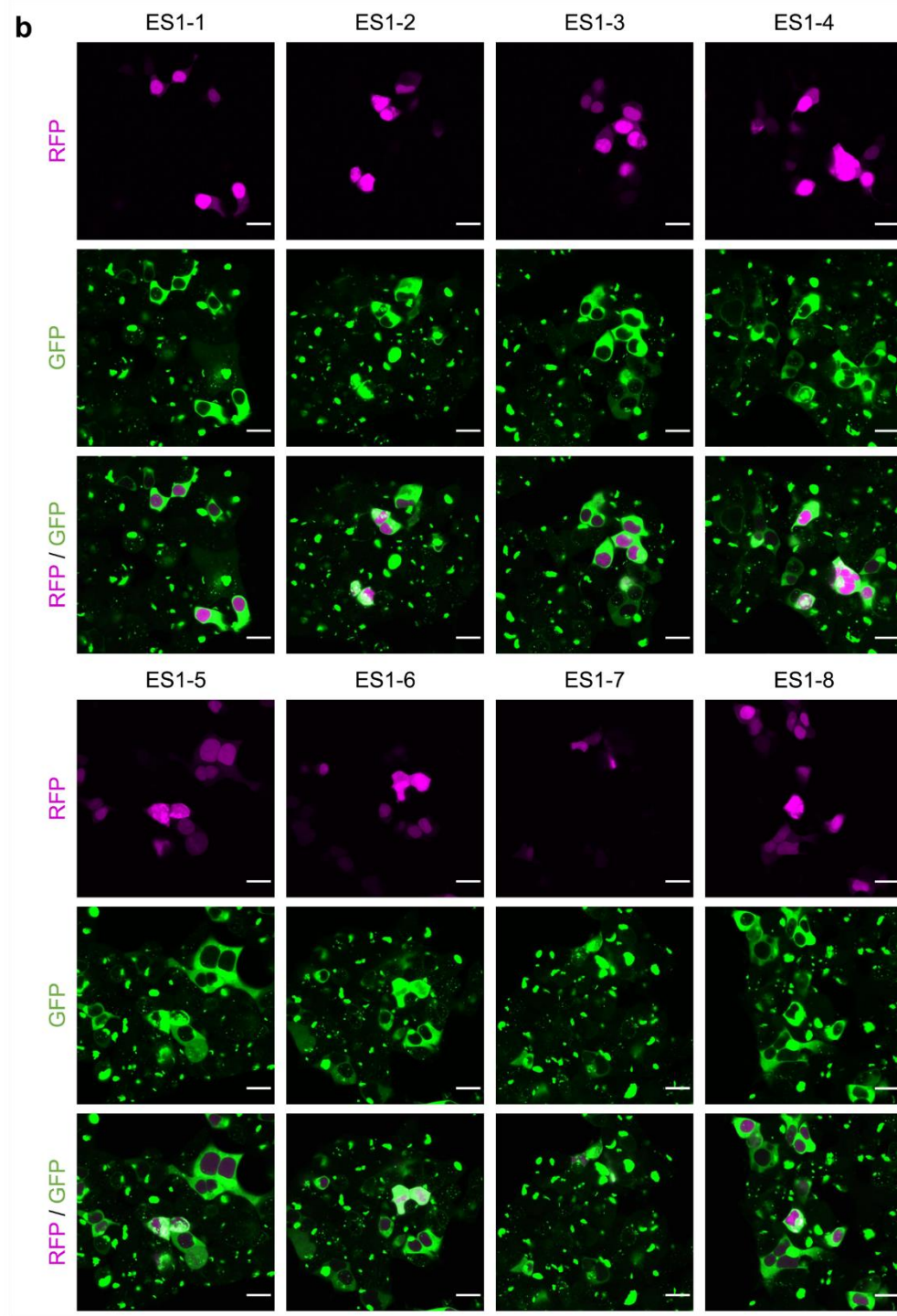

Supplement: Supplementary file 7 — Additional file 7: Supplementary Figure 7. Nuclear-cytoplasmic compartmentalization in ES1 cells. Control reporter cells (a) and ES1 cells (b) were transiently transfected with nuclear-cytoplasmic compartmentalization (NCC) reporter construct and imaged at 24 hours post transfection. The images were obtained from 8 different areas (160 μm2 for each), respectively. Faint dispersed (in the control) and strong aggregated tau signals (in ES1) were often overlapped with green fluorescent protein (GFP) signals derived from the NCC reporter. RFP, red fluorescent protein (in magenta). Scale bar, 20 μm. [file 12915_2021_1132_MOESM7_ESM.pdf]
